# Application of 3D Printing Technology to Produce Hippocampal Customized Guide Cannulas

**DOI:** 10.1523/ENEURO.0099-22.2022

**Published:** 2022-09-27

**Authors:** Maria Rosaria Tropea, Alberto Torrisi, Valeria Vacanti, Danilo Pizzone, Daniela Puzzo, Walter Gulisano

**Affiliations:** 1 Department of Biomedical and Biotechnological Sciences, University of Catania, 95123 Catania, Italy; 2 Oasi Research Institute-Istituto di Ricovero e Cura a Carattere Scientifico (IRCCS), 94018 Troina, Italy

**Keywords:** 3D printing, behavioral studies, brain cannulas, hippocampus, neuroscience method, open source apparatus

## Abstract

Implantation of guide cannulas is a widely used technique to access specific brain areas. Although commercially available, the need to personalize these implants and the high cost prompted us to design open-source customized devices taking advantage of 3D printing technology. Our cannulas consisted in a 3D-printed head mount designed according to the Paxinos coordinates to reach the CA1 area of the hippocampus. To cut guide cannulas to the proper length, we designed and realized an original 3D-printed linear motion apparatus. Polylactic acid thermoplastic polymer was used as printing material. Homemade or commercial cannulas were implanted in 4- to 6-month-old wild-type mice and intrahippocampal injections of amyloid-β peptide at different concentrations were performed. *In vivo* behavioral studies of novel object recognition indicated that results obtained with homemade versus commercial devices were comparable. Methylene blue injections and Nissl staining confirmed the correct localization of cannulas in the CA1 area of mouse hippocampus. Our method allows a fast manufacturing of hippocampal cannulas preserving the required precision at very low cost. Furthermore, this system can be easily modified to produce cannulas to target other brain areas. In conclusion, 3D printing might be used as a useful and versatile technology to realize open-source customized devices in neuroscience laboratories.

## Significance Statement

In the neuroscience field, several methods require a chronic access to specific brain structures. Although commercially available, the difficulties often encountered in terms of waiting time, high cost, and need to customize these devices to target different brain areas, prompted us to realize an open-source modular apparatus to produce guide cannulas exploiting 3D printing technology. Here we present a step-by-step procedure to realize hippocampal guide cannulas. The adequate manufacturing was validated by *in vitro* and *in vivo* experiments, and the advantages were compared with available commercial approaches. We believe that this work might be very useful to conduct research in all the fields where access to brain areas is needed.

## Introduction

The possibility of targeting specific brain areas is widely used in the neuroscience field, and several methods have been developed with the aim of administering drugs, delivering viral vectors, and installing electrodes or optical fibers. Among these, cannula infusion systems are widely used for acute and chronic drug delivery in the brains of animal models (Dong, 2018). The precise positioning of cannulas is granted by stereotaxic surgery that, relying on a three-dimensional coordinate system, allows targeting the brain area of interest with micrometric precision (Geiger et al., 2008). However, the increasing complexity of the scientific dilemmas requires customized solutions requiring very specific tools.

In this context, 3D printing technology has become an extremely useful resource (Vyavahare et al., 2020), especially after the expiration of fundamental patents, that previously slowed down its dissemination to a broader public (Hull and Arcadia, 1986). Concomitantly, new plastic materials have been developed, allowing the growth and spread of the application of 3D printing technology in biomedical industry, including various fields of medicine and neuroscience (i.e., dentistry, preoperative planning, prosthesis creation, drug discovery, medical training, and production of laboratory equipment; Baden et al., 2015; O’Brien et al., 2016; Dunham et al., 2018).

3D printing is an additive process that allows the physical realization of tridimensional objects under computer control. A computer-aided design (CAD) software is used to prototype the 3D model with accurate real-world dimensioning. The 3D project is then elaborated by a “slicer” software that cuts the model into a series of two-dimensional cross sections used by the machine to create the object in a layer-by-layer fashion. Among the different technologies, fused deposition modeling (FDM) is one of the most used additive processes in 3D printers in nonindustrial environments. FDM printers use a melt extrusion process where a solid thermoplastic material is fed through an electric motor into a heated liquefier or printing head. The latter is moved along the XY plane on top of a printing bed while depositing the melted plastic on it. After a layer is completed, the platform or the printing head is moved along the *z* axis by the thickness defined during the slicing process, allowing deposit of the following layer. This process is repeated in a layer-by-layer fashion until the object is completed (Masood, 2014; Penumakala et al., 2020; Vyavahare et al., 2020).

3D printing technology is commonly used in several laboratories to build a variety of objects (i.e., tube holders, Western blot pestles, and tools for behavioral studies). Recently, we wanted to explore the use of 3D printing to produce customized guide cannulas for intrahippocampal drug delivery in mouse models. In fact, although these devices are commercially available, the high costs, especially when several of them are needed for a set of experiments, the poor modularity, and the long waiting times if a customized nonstock length is needed, might represent a serious difficulty in ensuring laboratory efficiency. Based on these premises, our goals were as follows: (1) to produce cannulas guaranteeing high quality standards required for scientific experiments; (2) to obtain adequate results with minimum cost; (3) to realize a versatile system able to produce customized cannulas for different brain areas; and (4) to make our system usable by other laboratories.

## Materials and Methods

### 3D printer

All of the 3D-printed objects used for this work were realized by a customized version of the i3 FDM 3D printer (Prusa Research). The printer used an E3D V6 hot end (E3D) coupled with a Titan extruder (E3D) in a direct extrusion configuration. The *z*-axis movement was transmitted by 8 mm lead screws. Electronics consisted of Freaduino Mega 2560 plus Ramp 1.4. NEMA 17 Bipolar stepper motors (2.5 A, 1.8°/step) controlled by StepStick A4988 Drivers. All prints in this work were made with polylactic acid (PLA) filament (diameter, 1.75 mm).

### Hippocampal cannulas

The head mount was 3D printed in PLA with a 250 μm nozzle at 205° (210° for the first layer). Layer height was set at 50 μm, while the printing speed was set at 40 mm/s with an extrusion multiplier of 0.95. As a source of surgical steel, 26 gauge 80 mm spinal needles cut at the appropriate length were used.

Before production, we evaluated the following: (1) the length of the guide to reach the brain area of interest; (2) the interdistance between guides (corresponding to mediolateral distance × 2) to produce a double-sided guide cannula; and (3) the diameter of the guide based on the devices to be inserted (surgical steel guide). To calculate the guide length and the interdistance between guides, we referred to the Paxinos mouse brain atlas. For the guide diameter, considering the internal cannula used to infuse drugs, we chose 26 gauge spinal needles that were sufficiently large to make the internal cannula enter but prevented it from swinging.

Cannulas were photographed by a USB digital microscope, and length was analyzed by the Image J software.

### Linear motion-cutting system

The linear motion-cutting system was realized by assembling different components that were 3D printed in PLA. The detailed materials needed to realize the cutting apparatus is reported in Table 1. The list of required 3D-printed parts is reported in Table 2.

**Table 1:** Bill of materials for the cutting apparatus

Item	Description	Quantity	Cost (€)
3D printer	Any FDM with sufficient build volume	1	400–1500
Stainless steel rod	200–300 mm, Ø, 8 mm	2	10
Linear ball bearing	LM8UU Ø, 8 mm	3	5–10
Neodymium magnets	Cylindrical 10 × 2 mm	5	2
C- Clamps	Size depends on the mounting surface	4	20
Screws, nuts, washers and springs	Supplementary information	Supplementary information	30
Multitool	Dremel rotary tool or equivalent	1	50–100
Blade and blade support	Diamond coated	1	30
		Total	550–1700

The required hardware for building the cutting apparatus and start the cannula production. All costs are expressed in euros.

**Table 2:** List of the required 3D-printed parts for building the cutting apparatus

Item	Quantity	Production	Cost (€)
Bottom rail support	1	4 h 30 min	2.30
Top rail support	1	7 h	4.20
Multitool positioner	1	3 h 30 min	1.20
Multitool support	1	5 h	1.70
Multitool tightener	1	50 min	0.30
Multitool spacer	2	10 min (20 min total)	0.05 (0.10 total)
Base carriage	1	2 h 15 min	0.70
Length regulator base	1	1 h 50 min	0.65
Length regulator	1	1 h 45 min	0.50
Centering support	2	10 min (20 min total)	0.025 (0.05 total)
Cannula support: hippocampus	1	40 min	0.20
Cannula support: cover	1	15 min	0.10
Cutting length: regulator handle	1	20 min	0.05
Carriage handle	1	1 h 20 min	0.30
Total		30 h	12.35

All production times are derived from the following printing parameters: speed = 60 mm/s; layer height = 200 μm; infill = 20%; nozzle diameter = 400 μm. Different parameters will influence printing times. Reported costs are an estimate, based on that of generic PLA. All costs are expressed in euros.

### Design files

Detailed step-by-step instruction to realize the cutting system can be found in Extended Data Figure 1-1. Standard for the Exchange of Product Data (STEP) files, Stereo Lithography interface format (STL) files, and technical drawings to reproduce our system (guide cannulas and cutting system) can be found in Extended Data Figure 2-1.

**Figure 1.**
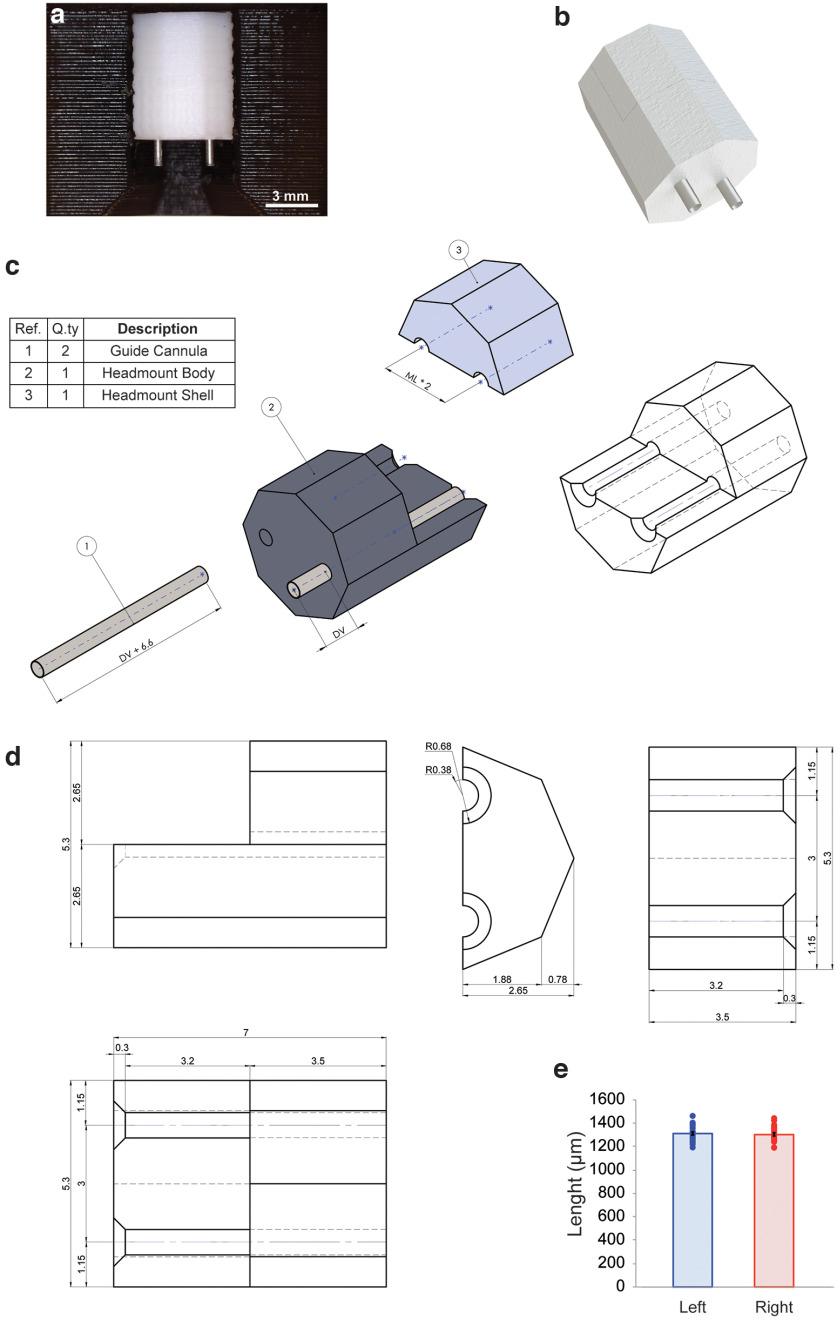
Representation of head mount and guides. ***a***, Photograph of a cannula of our production. ***b***, Rendered image of an assembled cannula. ***c***, Exploded isometric view of the components and key parameters of a guide cannula. ***d***, Technical drawing of the head mount, including all of the relevant dimensions. All measures are expressed in millimeters. ***e***, Left and right guides measured in 26 cannulas have the correct expected length of 1330 μm. Data are expressed as mean ± SEM.

10.1523/ENEURO.0099-22.2022.f1-1 Figure 1-1 Detailed step-by-step building guide for the linear motion-cutting system. Download Figure 1-1, DOCX file.

**Figure 2.**
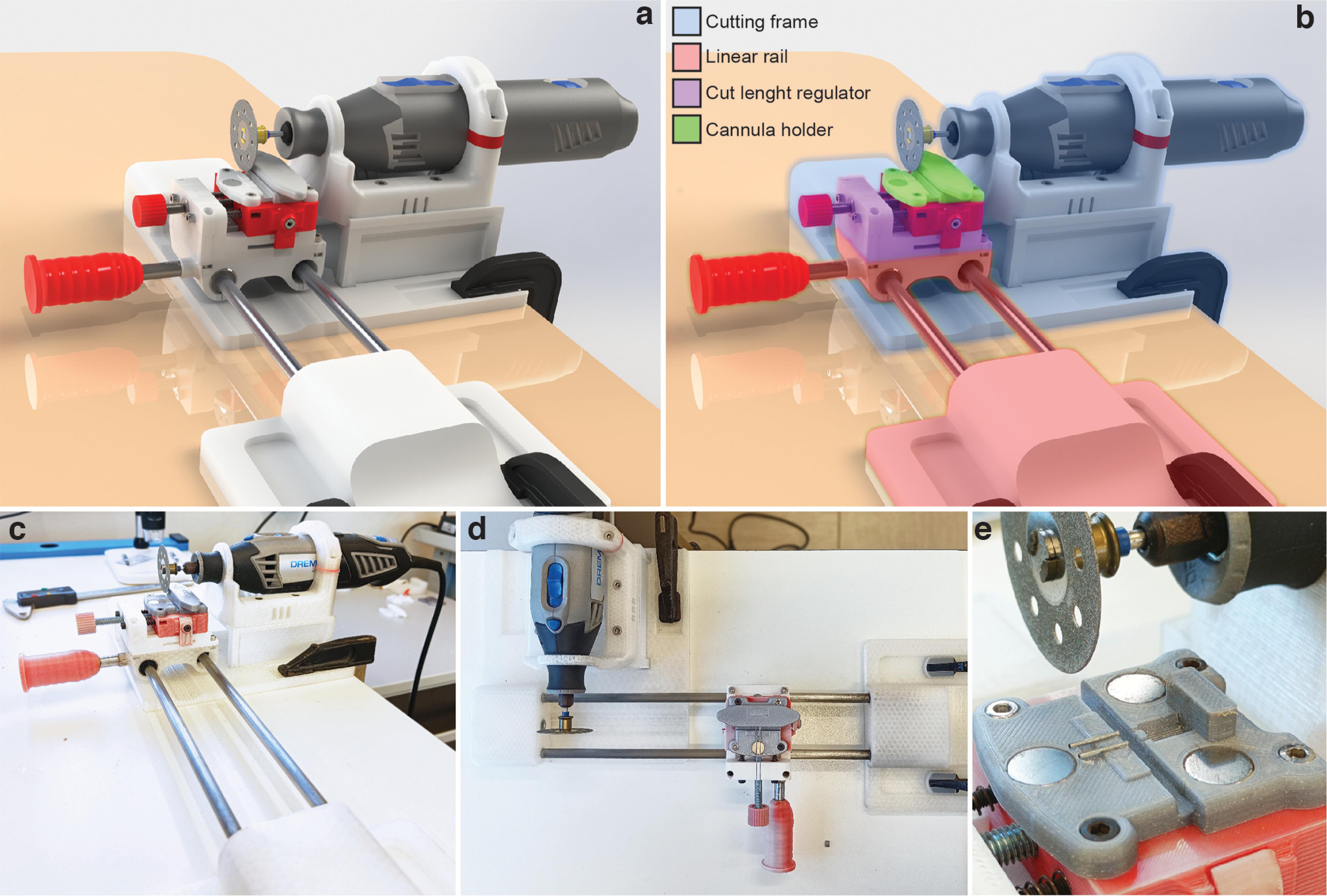
Linear motion-cutting system. ***a***, Rendered representation of the cutting system. ***b***, Colored highlight of the single components, as follows: (1) light blue, the cutting frame, formed by a base to host the final part of the linear rail and a support for the handheld rotary tool (Dremel); (2) pink, the linear rail, consisting of two 8 mm steel bars held by 3D-printed components to form a sliding carriage; (3) violet, the cut length regulator allowing determination of the length of the guides; and (4) green, the cannula holder placed on top of the carriage to host and hold the head mount in place during the cutting process. ***c–e***, Photographs with front, top, and closeup views of the actual apparatus built in our laboratory.

10.1523/ENEURO.0099-22.2022.f2-1 Figure 2-1 *.Stl files, *.STEP files, and technical drawings. Download Figure 2-1, ZIP file.

### Validation of the homemade cannulas

#### Animals

Experiments were performed on sex-balanced 4- to 6-month-old C57BL6/J wild-type mice (RRID:IMSR_JAX:000664), obtained from a breeding colony kept at the University of Catania. Mice were maintained with a controlled temperature (21 ± 1°C) and humidity (50%) on a 12 h light/dark cycle, with *ad libitum* access to food and water. All experiments involving animals were conducted in accordance with the guidelines laid down by the European Community Council (2010/63/EU) and the ARRIVE (Animal Research: Reporting of In Vivo Experiments) guidelines. The experimental protocols were approved by the University Institutional Animal Care and Use Committee (Protocols #327/2013-B and #119–2017-PR). The experiments complied with the ARRIVE guidelines and were conducted to minimize the pain and suffering of the animals.

#### Surgical procedure

Cannula implantation was performed as previously described (Gulisano et al., 2019; Leggio et al., 2019). Aft er anesthesia with intraperitoneal injection of tiletamine plus zolazepam (60 mg/kg) and medetomidine (40 μg/kg), mice were implanted with our hippocampal homemade guide cannulas or commercial ones according to the following coordinates: anteroposterior = −2.46 mm; mediolateral (ML) = ±1.50 mm, dorsoventral (DV) = 1.3 mm. Cannulas were fixed to the skull with acrylic dental cement (Rely XTM, Unicem, 3M), and mice were allowed to recover for a minimum of 6–8 d after surgery.

#### Nissl staining

Mouse brains were removed and fixed in 4% paraformaldehyde in 0.1 mol/L phosphate buffer solution, pH 7.4. Then, serial coronal sections of the brains were cut at 80 μm thickness. Sections in the range between ±240 μm from the cannula insertion site were stained with Nissl staining (0.1% thionin), a histologic method classically used to assess morphology and general cell distribution, and visualized by light microscopy at 4× magnification (Gulisano et al., 2019). In another set of experiments, methylene blue was infused, and the brains were then removed and freshly cut to visualize the marker.

#### Amyloid β preparation and infusion

Oligomeric amyloid β 42 (Aβ42) was prepared as previously described (Stine et al., 2003) to reach a final concentration of 200 nm or 200 pm, which are known to exert opposite effects on memory processes (Gulisano et al., 2018). Aβ or vehicle was bilaterally infused into the hippocampus in a final volume of 1 μl over 1 min with a microsyringe connected to the cannulas via polyethylene tubing. During infusion, animals were handled gently to minimize stress. After infusion, the needle was left in place for another minute to allow diffusion.

#### Novel object recognition

Mice were divided in the following experimental groups and treated by intrahippocampal injections through our homemade cannulas or commercial devices: (1) eight mice treated with 200 pm Aβ; (2) eight mice treated with 200 nm Aβ; and (3) eight mice treated with vehicle.

Novel object recognition test was performed as previously described (Gulisano et al., 2019). The arena was a white plastic box, and objects were 3D printed in PLA (Gulisano et al., 2018). Mice underwent 3 d of habituation to freely explore the for 10 min the arena (day 1) or two objects randomly chosen among our collection (days 2–3). During the fourth day, mice underwent the intrahippocampal treatment 20 min before the training phase (T1), consisting in a 10 min exploration of two identical objects inserted into the arena. After 24 h, during the testing phase (T2), one of the objects explored in T1 was substituted with a novel object, and mice were allowed to explore for 10 min. After each trial, the box and the objects were cleaned with 70% ethanol and dried with absorbent paper.

The time spent exploring the objects was scored using a personal computer by an experimenter who was blinded to the conditions tested. We measured the discrimination index (D; exploration of novel object minus exploration of familiar object/total exploration time) and total exploration time. We excluded from the analysis mice with a total exploration time <5 s.

### Statistics

Statistical analysis was performed by using different tests, based on preliminary analyses of normal distribution. One-way ANOVA with Bonferroni’s *post hoc* correction was used to analyze the discrimination index and total exploration time in different experimental conditions in animals implanted with commercial or homemade cannulas. Two-way ANOVA using treatment and cannula type as variables was used to compare results obtained in commercial versus homemade cannulas. One-sample *t* test was used to compare obtained length with expected length of guides (1300 μm). Systat 9 software was used for statistical analyses. Data were expressed as the mean ± SEM. The level of significance was set at *p* < 0.05.

### Data availability

We have made our device available under an open source patent in Creative Commons.

## Results

### Realization of intrahippocampal cannulas

Product validation, from design to realization, had the sole aim of easily creating a quality product to be used in our laboratory. No industrial or commercial purposes were considered.

The cannula consisted of a 3D printed head mount in which surgical steel guides were inserted and cut at the appropriate length (Fig. 1*a*). We first designed the double guide cannula to reach bilaterally the dorsal hippocampus (Fig. 1*b*). The head mount had an octagonal prism shape and an internal allocation space for the two guide cannulas (length, 6.6 mm; diameter, 0.65 mm) with a 1.5 mm interspace (Fig. 1*c*,*d*). Before its assembly, the head mount consisted of two parts (i.e., a body and a shell), a design chosen to allow an easy placement of the guides and the possibility of fixing them in the correct position. Guides were easily placed into the head mount, fixed in the correct position, and cut to the appropriate length by a customized cutting apparatus. The length of the left guide (1308.76 ± 12.6 μm) and right guide (1299 ± 12.4 μm) were equal to the expected length (1300 μm; one-sample *t* test; left side: *t*_(25)_ = 0.696, *p* = 0.493; right side: *t*_(25)_ = 0.073, *p* = 0.942; Fig. 1*e*). The patency of both guide cannulas was verified after cut. To assess it, a smaller guide was inserted into both cannulas, ensuring that it passed all the way through. The production success ratio in terms of patency was 90%.

### Realization of the cutting apparatus

The prototype of the cutting apparatus was designed to obtain steel guides with a uniform and proper length. It consisted of a linear motion carriage that could be pushed straight to a cutting blade of a fixed rotary tool (Fig. 2*a–e*; see Extended Data Figs. 1-1 and 2-1 for details of cutting apparatus realization).

**Figure 3.**
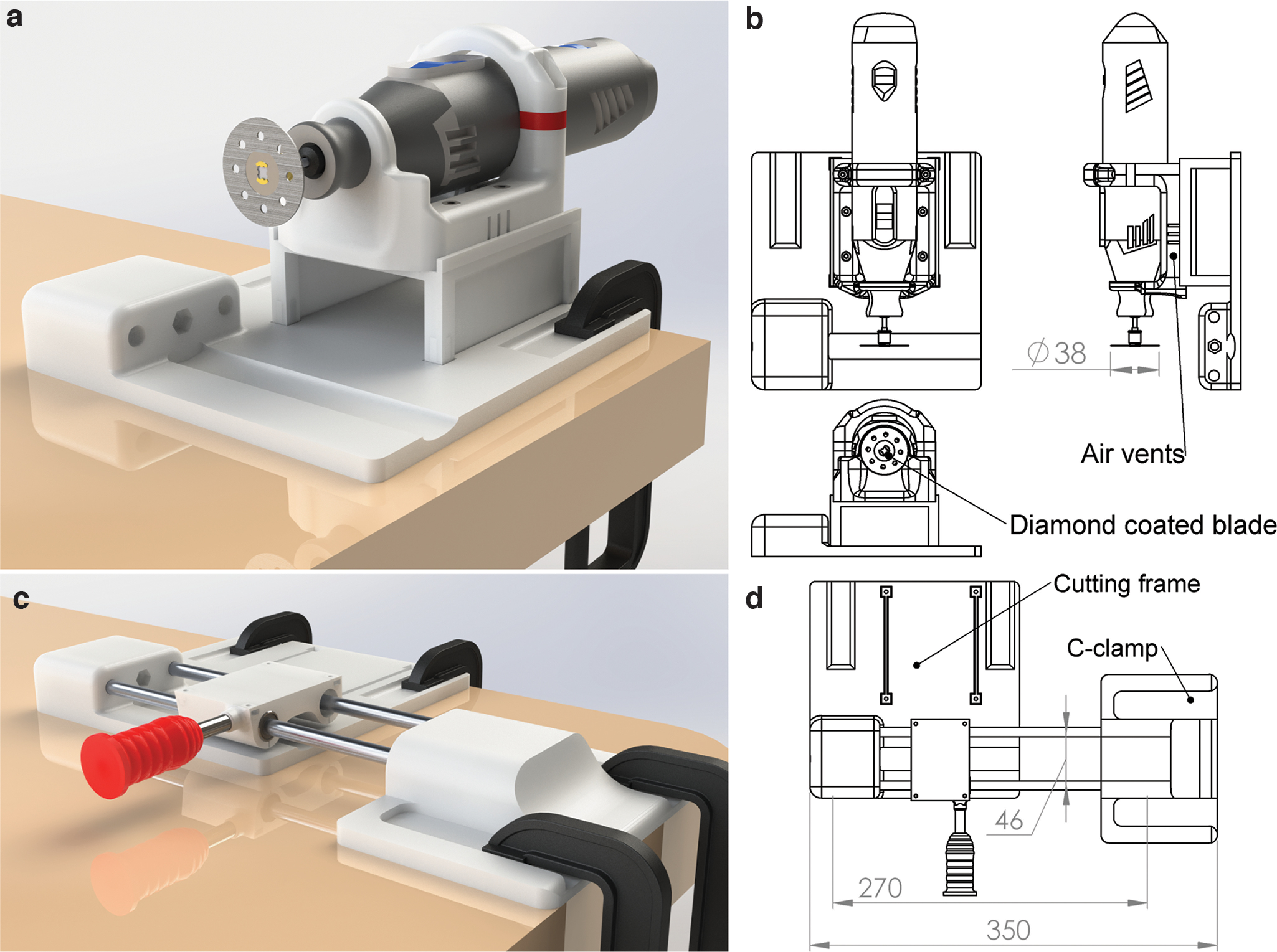
Descriptive images of the cutting frame and the linear rail. ***a***, Rendered image of the isolated cutting frame clamped to the table. ***b***, Schematic drawing of the cutting frame. The rotary tool support is mounted on the base and fixed to a structure that can be moved to modify the position of the cutting blade and host air vents to avoid overheating. The rotary tool is equipped with a 38 mm circular diamond-coated blade. ***c***, Rendered image of the linear rail and the base of the cutting frame, both clamped to the table. ***d***, Schematic drawing of the linear rail that is composed of two parallel 8 × 270 mm bars with a 46 mm interaxial distance. The carriage consists of a base with two blind holes hosting three LM8UU linear ball bearings, two on one side and one on the other side to ensure a straight alignment of the 8 mm bars. The base is equipped with a 3D-printed handle to move the carriage back and forth along the linear rail. Another carriage is mounted on the top of the base by four socket head cap M3 screws.

After realization, we first evaluated the stability of the cutting frame and found that it was secured by the wide surface area and the presence of two grooves hosting C-clamps to fix the structure to a table. This arrangement attenuated vibrations caused by the rotatory tool (Fig. 3*a*,*b*). The rotary tool support, mounted on the base and fixed to a movable structure, allowed modification of the position of the cutting blade (Fig. 3*a*,*b*).

The parallel positioning of the bars of the linear rail was granted by hosting their extremities in the base of the cutting frame and, on the opposite side, in a 3D-printed support clamped to the table (Fig. 3*c*,*d*).

**Figure 4.**
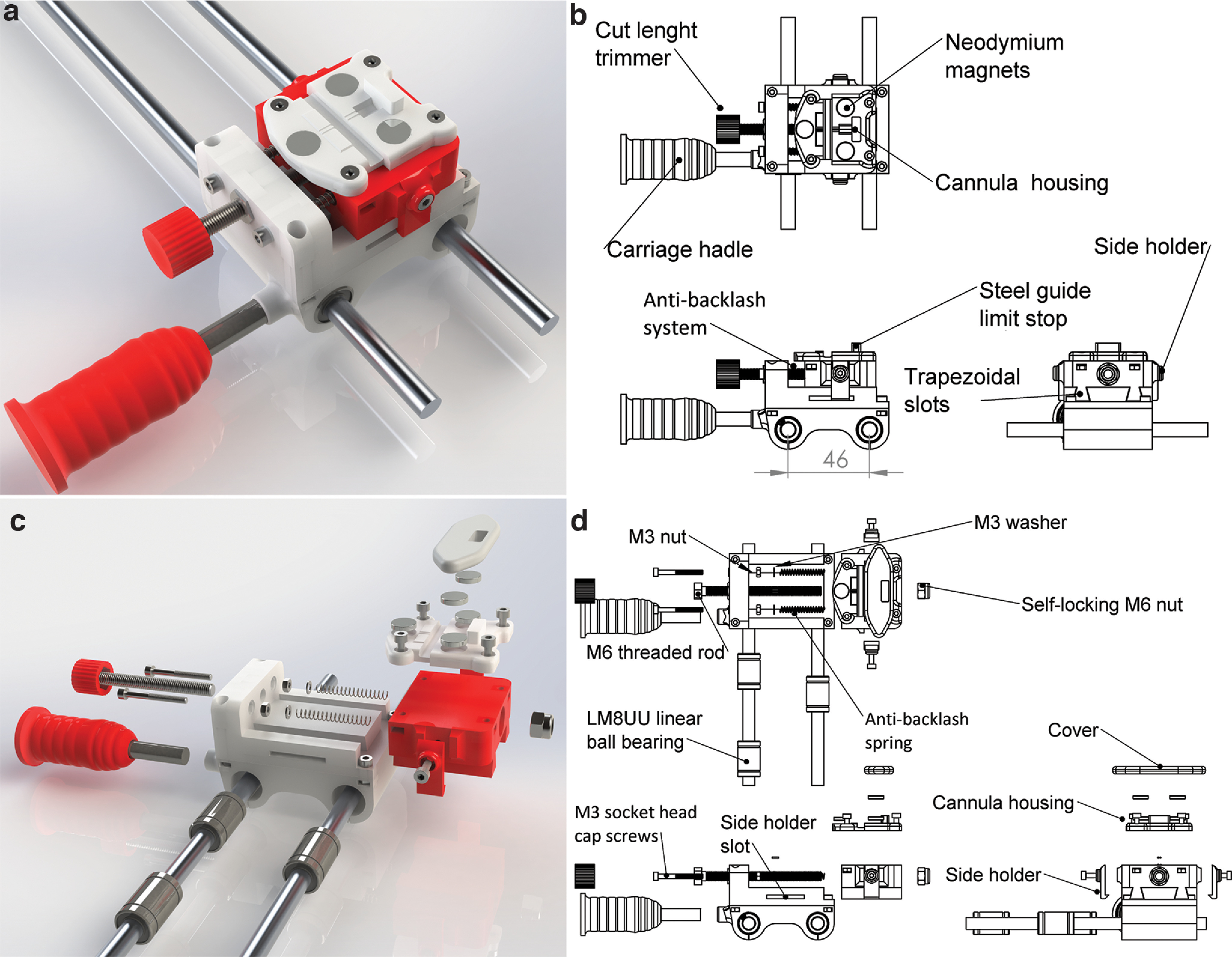
Cutting length regulator. ***a***, Rendered image of the cutting length regulator, consisting of two main parts, both 3D printed. ***b***, Schematic drawing of the cutting length regulator. The moving part is inserted into its base by two trapezoidal slots. ***c***, Rendered exploded view of the cutting length regulator. ***d***, Schematic drawing of the exploded view in ***c***. The displacement of the carriage is granted by the following two components: an M6 threaded rod, passing through the base and the carriage, with a self-locking M6 nut; an antibacklash system composed of two M3 socket head cap screws supporting two springs pushing from the surface of the base to the inside of the carriage. The carriage can be moved by turning the screw and is held in place by the tension of the springs. Two holders are placed on the side of the carriage fixing it to the base.

The carriage could be easily moved back and forth along the linear rail by a 3D-printed handle (Fig. 4*a*,*b*). The system was held in place by the tension of the springs and stabilized by two holders placed on the side that fixed the carriage to the base (Fig. 4*c*,*d*).

**Figure 5.**
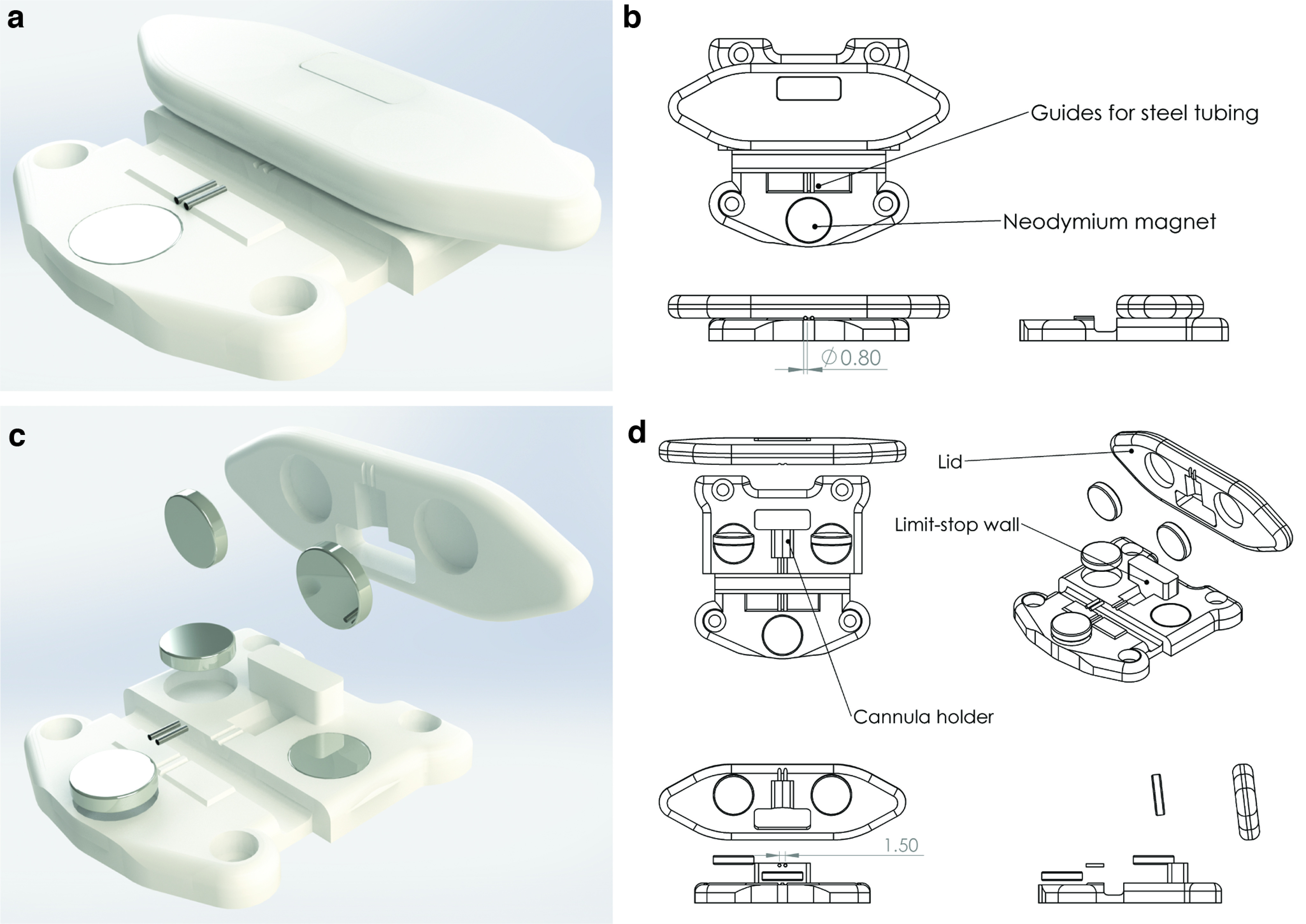
Cannula holder. ***a***, Rendered image of the cannula holder. ***b***, Schematic drawing of the cannula holder formed by a base and a lid. The base, designed to host the cannula head mount, shapes its octagonal form and presents with grooves for the guide cannulas to stay in place. ***c***, Rendered exploded view of the cannula holder. ***d***, Schematic drawing of the exploded view in ***c***. The cannula holder hosts 10.2 × 2.2 mm cylindrical neodymium magnets. Two magnets are inserted on the base of the cannula holder, two on the lid, and one in front of the guides.

**Figure 6.**
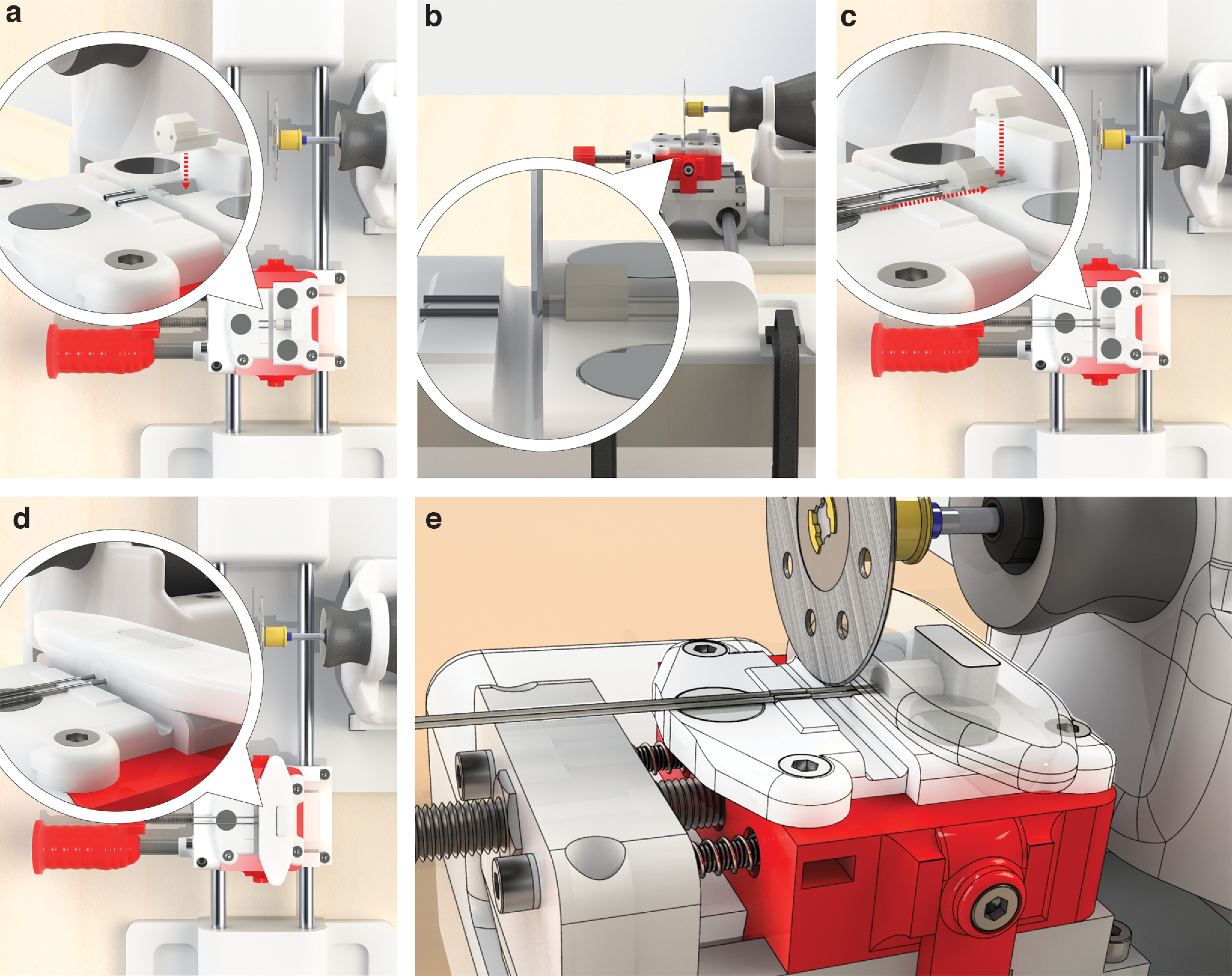
Illustrated step-by-step process of the cutting procedure. ***a***, Positioning of cannula body in its holder with the open side facing the end limit wall. The head mount body should be placed into its housing shaped in the cannula holder oriented as shown in the insert. ***b***, Regulation of the cutting length. Carriage is pushed underneath the blade (multitool turned off), and its relative position is adjusted to the blade. For optimal positioning, the blade is as close as possible to the cannula without touching it. ***c***, Positioning of the steel guides and the head mount shell. The carriage is placed back into a safe position. Steel tubes (spinal needles) are inserted into the head mount through the appropriate guides until they reached the limit stop represented by the wall behind the cannula. A drop of superglue is placed on the cannula body and is covered by the shell part of the cannula to let the head mount be assembled. ***d***, Positioning of the magnetic lid to ensure a tight compression on the head mount ensured by the magnets. ***e***, Cutting of the steel guides at the appropriate length. The rotary tool (Dremel) is turned on at ∼5000 rpm. The main carriage is pushed to the rotating blade cutting the guides. After cutting, the carriage is moved back to the starting position, the rotary tool is stopped, and the lid is removed to extract the cannula.

The cannula holder was adequate to host the cannula head mount. The cylindrical neodymium magnets (Fig. 5*c*,*d*) were able to keep guides in place during the cutting process that is described in detail in Figure 6.

### Homemade cannulas reached the CA1 area of the hippocampus

**Figure 7.**
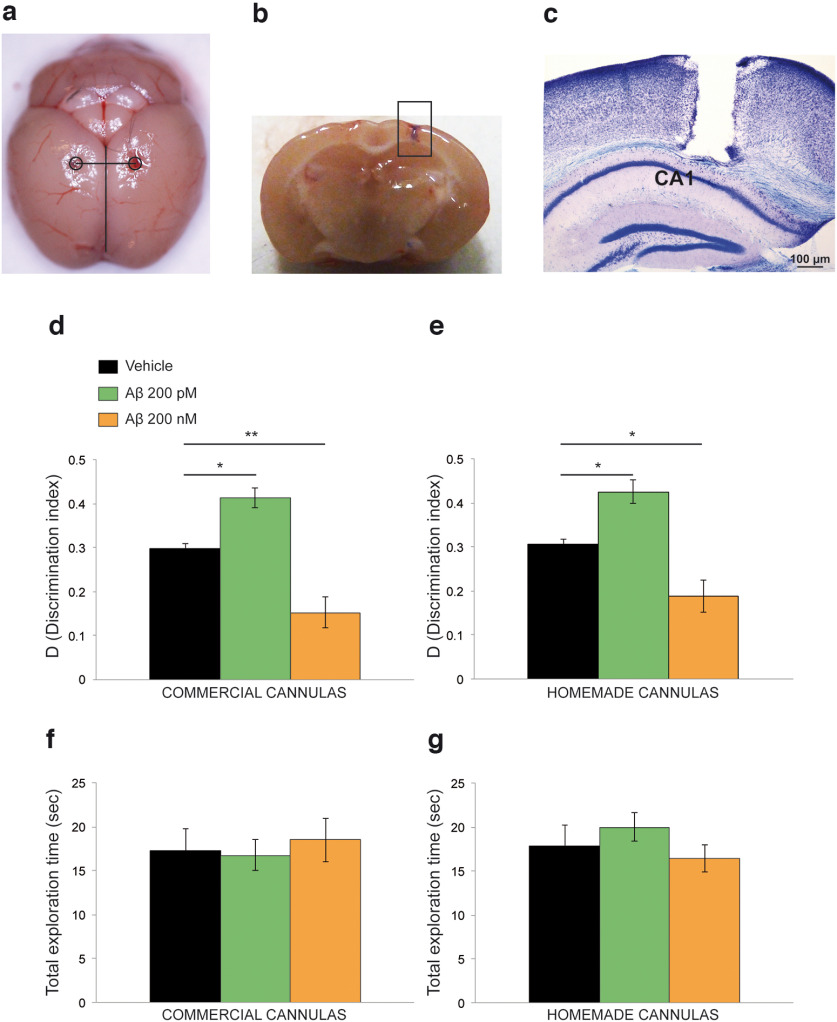
Intrahippocampal cannula validation. ***a***, Mouse brain after cannula removal. Guide holes are equidistant from the interhemispheric fissure. ***b***, Injections of methylene blue in the right hemisphere reaching the CA1 area. ***c***, Nissl staining of the coronal section of a mouse left hemisphere. The cannula lesion is visible above the hippocampus. ***d***, In mice implanted with commercial cannulas, intrahippocampal injections of 200 pm Aβ increased the discrimination index, whereas injection of 200 nm Aβ impaired it. ***e***, The same results are obtained in mice infused through our homemade cannulas. ***f***, ***g***, Total exploration time is not modified by treatment in mice injected by commercial (***f***) or homemade (***g***) cannulas. *p < 0.05; **p < 0.01. Data are expressed as the mean ± SEM.

After production, we evaluated whether guides effectively reached the target area (i.e., the hippocampus). We observed the fresh brain to exclude macroscopic alterations of the tissue because of the procedure or the reaction between PLA and acrylic cement. The mouse brain that resulted was intact except for the two entrance holes of the guide cannulas (Fig. 7*a*). Methylene blue infusion showed that the marker reached the CA1 area of the hippocampus (Fig. 7*b*), and Nissl staining confirmed the correct position of the homemade cannula (Fig. 7*c*).

### Results of *in vivo* behavioral studies obtained with homemade cannulas were comparable to commercial ones

To functionally validate our cannulas, we performed *in vivo* studies to evaluate whether data obtained with our homemade cannulas were comparable to those from commercial devices. To this end, we performed intrahippocampal injections of different concentrations of Aβ and studied its effects on the novel object recognition behavioral task. Analyses of D confirmed that 200 pm Aβ improved while 200 nm Aβ impaired recognition memory in both mice treated by commercial (one-way ANOVA: *F*_(2,21)_ = 21.282, *p* < 0.0001; Bonferroni’s correction: *p* = 0.025 between vehicle and 200 pm Aβ; *p* = 0.005 between vehicle and 200 nm Aβ; Fig. 7*d*) or homemade cannulas (one-way ANOVA: *F*_(2,21)_ = 14.742, *p* < 0.0001; Bonferroni’s correction: *p* = 0.038 between vehicle and 200 pm Aβ; *p* = 0.04 between vehicle and 200 nm Aβ; Fig. 7*e*), thus confirming the efficiency of our customized system. Total exploration time was comparable between the two groups in the three different experimental conditions (i.e., vehicle, 200 pm Aβ, and 200 nm Aβ; one-way ANOVA: *F*_(2,21)_ = 0.116, *p* = 0.891 for commercial cannulas; *F*_(2,21)_ = 0.634, *p* = 0.541 for homemade cannulas; Fig. 7*f*,*g*). Two-way ANOVA, using treatment and cannula type as variables, confirmed that there were no differences in results obtained through commercial or homemade cannulas when analyzing D (*F*_(2,42)_ = 0.119, *p* = 0.888) or total exploration time (*F*_(2,42)_ = 0.575, *p* = 0.567).

### Production time and costs

After validation, we calculated possible advantages in terms of money and time saving. The price to purchase the required hardware ranges from 600 to 1500 €, including the 3D printer and the multitool (Table 1). To produce the cutting apparatus, most of the components can be 3D printed in ∼30 h of total print time, costing <15 € (if using PLA as printing material; Table 2). The apparatus can be assembled by a nonexperienced user in ∼2 h. Regarding the cannula production cost and time, the production process can be subdivided into the following three steps: (1) printing; (2) assembly and cut; and (3) validation.

#### Printing

A batch of 40 head mounts (including the shell) can be printed in ∼6 h and 30 min at a cost of ∼0.17 € using the following high-resolution printing parameters: speed, 30 mm/s; layer height, 50 μm; infill, 20%; nozzle diameter, 250 μm. Costs and printing times for differently sized batches can be found in Table 3.

**Table 3:** Production costs and times for differently sized cannula batches

Cannulas (*n*)	Estimated print time	Print cost	Estimated time for assembly, cut and validation	Total cost (€)
1	11 min		4	0.3
5	50 min	0.02	20	1.52
10	1 h 40 min	0.04	40 min	3.04
20	3 h 15 min	0.08	1 h 20 min	6.08
40	6 h 30 min	0.17	2 h 40 min	12.17
50	8 h	0.21	3 h 20 min	15.21
100	16 h	0.4	6 h 40 min	30.4

Total cost includes the price of the steel guides. All costs are expressed in euros.

#### Assembly and cut

Assembly of the steel guides with the head mount can be performed as previously shown (Fig. 6). 26 gauge spinal needles cost 0.02 €/mm. Considering that the length of a hippocampal steel guide is 6.6 mm, it results in a cost of 0.15 € each (thus, 0.30 € for a pair). For a batch of 40 cannulas, the cost in terms of material for the steel guides is ∼12 €. The assembly and cutting time for each piece varies with the experience of the user, but after an initial training every piece can be assembled and cut in <3 min.

#### Validation

In this last step, it is important to verify that all the needles stay in place even if pushed and that both guides are patent. The latter can be done by inserting a smaller needle into the guides to ensure that it passes through (in our case, the internal disposable guide of the spinal needles was used). Testing a cannula should require no more than 1–2 min.

In conclusion, a batch of 40 cannulas can be produced at a cost of 12.20 €, in ∼8 h (printing, 6 h; assembly time, 2 h; Table 3, different batch sizes).

## Discussion

In this article, we have shown how to realize customized cannulas to be implanted in animal models for injections of substances into specific brain areas. In particular, we have targeted the hippocampus and produced cannulas aimed to reach the CA1 area. The brain was not substantially damaged by our homemade cannulas, except for the lesion because of implantation, and positioning was correct as shown by methylene blue injections and Nissl staining. We have chosen to validate our system by injecting Aβ, a peptide known to be involved in Alzheimer’s disease pathogenesis and able to modify hippocampal function, depending on the dose (Gulisano et al., 2018). The results we have obtained through behavioral studies showed the efficacy of our method and confirmed that the experimental outcome is ensured.

To print all the objects used in this work, we have chosen PLA. Indeed, although a wide range of thermoplastic materials can be used based on their characteristics of resistance, simplicity of use, and biodegradability, PLA has the following characteristics that makes it the best option for applications in the biomedical field (Pawar et al., 2014; Matos et al., 2019): (1) it is degradable as its synthetic raw materials do not come from petroleum; (2) it offers good biocompatibility as proved by its use as medical suture material or drug sustained-release material; (3) because of the low melting point, its dimensional precision is more controllable; and (4) it presents very low decline in toughness in the long-term use (Liu et al., 2019). Possible disadvantages are represented by the fragility of PLA 3D-printed objects and the limited possibility of postprinting finishing (Gordeev et al., 2016). Even if it has been previously shown that PLA, when exposed to some chemical substances, can induce inflammatory reactions (Ramot et al., 2016) and, in the SNc, astrocyte and microglia activation, resulting in neural degeneration (Lewitus et al., 2011), in our conditions PLA was used to print the head mount that does not have any direct contact with the injected substances or the brain tissue.

When choosing a source for surgical steel guides, we opted for a cost-efficient solution represented by spinal needles because of their easy availability and certified use for medical purposes. However, for larger-scale productions it might be more efficient to order surgical steel tubing in larger quantities directly from specialized suppliers. The diameter of the spinal needle should be chosen based on the internal cannula used to inject drugs or in relation to the specific application (i.e., optical fibers). For our implementation, we used a 26 gauge needle with an 80 mm length allowing production of ∼53 guides (i.e., 26 double-sided cannulas).

3D printing can also be exploited to realize very low-weight implants that allow multiple electrode recordings and/or fiber optics for optogenetics in a more efficient way, as previously demonstrated (Voigts et al., 2013; Harris et al., 2017).

It is important to specify that, here, we have shown how to produce bilateral straight cannulas targeting the dorsal hippocampus. The design of our cutting apparatus does not allow production of angled cannulas, and this might represent a limitation in cases where such approach is required to access certain brain areas. However, the availability to the source files and drawings of the project, and the modularity of the apparatus might allow researchers to modify only a fraction of the parts composing the device to produce customized angled cannulas based on specific needs. The possibility of customized production can also facilitate the development of new tools that are not available in the market. For example, the experimenter can insert other devices (e.g., optic fibers, biosensors, or electrodes) or couple them with guide cannulas for drug infusion. In this case, a modification of the head mount is required to host multiple items within its body. Electrophysiological studies in behaving rodents might also benefit from personalized head mounts designed to ameliorate implantations of electrodes. For example, it is possible to include a hole that can be used as a reference point for an easier positioning of electrodes during the surgical procedure. When targeting a deeper structure, an additional steel guide as a housing device might be inserted to avoid bending the electrode during the implantation. To our knowledge, the cannula implantation should not interfere with electrophysiological recordings, as shown in previous works (Harris et al., 2017; Hernan et al., 2017). In fact, the head mount is made of PLA that falls into dielectric category in terms of electrical conductivity and, according to the literature, it should not alter electrophysiological recordings; indeed, to achieve electric conductivity, PLA must be modified with carbon nanofillers such as nano-graphite, carbon nanotubes, or graphene (Guo et al., 2019). On the other hand, guides were made of surgical stainless steel (SAE 440 and SAE 420), a high carbon steel alloyed with chromium, which has slight magnetic properties. Even if its influence should be negligible, depending on the required characteristics, different kinds of stainless steel tubing with lower a magnetic permeability (e.g., austenitic stainless steel, which is compatible with magnetic resonance imaging apparatuses) can be used (see ASTM International specifications: https://www.astm.org/f0899-20.html). Thus, printing with alternative materials might be considered to overcome this possible issue, but, in any case, a validation procedure is recommended before starting implementation of electrophysiological recordings with our device.

In addition to the possibility of customizing the devices, a relevant advantage of our system is represented by cost reduction. In fact, a single purchased cannula usually costs ∼10–14 €, while the materials needed to produce a homemade cannula cost ∼0.25 €, thus allowing a 98.3% saving. Even if this cost does not include the 3D printer purchase, in recent years prices are becoming affordable (from 250–700 €, depending on the model), thus allowing amortizing the start-up costs in a short period of time, especially considering that 3D printing can be used for several purposes (Baden et al., 2015; Coakley and Hurt, 2016; Urban, 2016; Velcescu et al., 2019).

Furthermore, the cost remains much lower if compared with the commercial products, even when considering the production time. In fact, once manufactured, the cutting carriage time required by an operator to produce one cannula (head mount printing plus assembly) is ∼3–4 min.

In conclusion, the use of 3D printing technology is increasing in several biomedical fields. In neuroscience research, it has been highly exploited for the possibility to create customized devices that are not available in the market, to modify and make objects more functional for a certain need, and to reduce costs. The open-source concept, often pursued by the users of these technologies, has induced an increasing number of laboratories to share the blueprints for their models, and several freeware and user-friendly CAD programs are being released, making the approach toward this field easier. Hence, this investment would make it possible to have a permanent useful tool for different applications in a neuroscience laboratory (Chen et al., 2017).

## Synthesis

Reviewing Editor: Laura Bradfield, University of Technology Sydney Decisions are customarily a result of the Reviewing Editor and the peer reviewers coming together and discussing their recommendations until a consensus is reached. When revisions are invited, a fact-based synthesis statement explaining their decision and outlining what is needed to prepare a revision will be listed below. The following reviewer(s) agreed to reveal their identity: NONE. Note: If this manuscript was transferred from JNeurosci and a decision was made to accept the manuscript without peer review, a brief statement to this effect will instead be what is listed below.

Dear Dr. Puzzo and authors,

Thank you for submitting your manuscript to eNeuro. Overall, the reviews were mainly positive, but the manuscript generated a lot of discussion upon consultation. Although the levels of enthusiasm were unequally distributed among the reviewers and editors, on balance it was decided that yours was a technologically sound manuscript that provided a guide to producing tools (cannulas) that are cheaper than those commercially available, and that will hopefully improve standardisation and reproducibility between labs. Therefore, we decided it did warrant publication in eNeuro, but only if the following points are adequately addressed. In particular, it is necessary to address the following:

1. There is concern that the approach will not allow for implanting angled cannulas for bilateral injections, or two cannulas in different brain areas, either angled or straight, for a disconnection experiment. If this is the case, the authors need to acknowledge in the discussion that their approach is limited to two straight bilateral cannulas, and that this limits the broader application of the approach. If this is not the case, and the procedures can indeed be modified to be used with angled cannulas, or cannulas that are implanted in different brain regions, could the authors please provide additional evidence of this (e.g. photomicrographs of the cannulas, and of cresyl-violet stained tissue of where they have been implanted) and provide detail how the procedure may need to be modified to allow for this.

2. There needs to be a section added to the discussion a section regarding how your system could be used for more complex cannula implants (as mentioned in point 1) but also the system for tubing of other kinds. Reviewer #2 questioned whether cannula implantation might impact electrophysiological recording, but it was suggested that printing with an alternative material could overcome this issue. Please could this be mentioned. More importantly, however, this concern should be additionally addressed by following the suggestion of reviewer #1 that the following be included: “the actual design files that a lab would modify to make a different 3D-printed canula holder, as well as modifying the jig for different gauges of tubing and different lengths.”

Please also address any additional points raised by the reviewers in the following reviews:

Reviewer #1

There are two significant improvements that could be made to the manuscript. First, critically, I would like to see at least one photo of the actual system. Otherwise, it raises concerns that it was not actually made as the renders depict and that results might thus be not directly applicable. Second, it is needed to share the actual design files in addition to the STL versions. This allows for users to make modifications without needing to redesign the whole system.

Reviewer #2

In this study, authors developed a 3D-print method which can be used to design open-source customized cannulas. To test its effectiveness, authors conducted in vivo behavior studies. Results show that the developed cannula is comparable with commercial counterparts. Another important benefit from this study is that the developed method can help reducing the cost of making guide cannulas significantly. Overall, this is an interesting study and methods and my feeling is, product from this study should be useful to the field.

I only had a general concern about the study:

Will the cannulas implantation impact the electrophysiology signal recording? Like LFP recording. If so, authors need to compare this impact with commercial one, like they did in the manuscript.
