# Supplementary material for: Application of 3D Printing Technology to Produce Hippocampal Customized Guide Cannulas
Source: eNeuro. 2022 Sep 27;9(5):ENEURO.0099-22.2022. doi: 10.1523/ENEURO.0099-22.2022 (PMC9522464; doi:10.1523/ENEURO.0099-22.2022)
Supplement: Figure 1-1 — Detailed step-by-step building guide for the linear motion-cutting system. Download Figure 1-1, DOCX file. [file enu-eN-MNT-0099-22-s01.docx]

# LINEAR MOTION CUTTING SYSTEM - BUILDING GUIDE


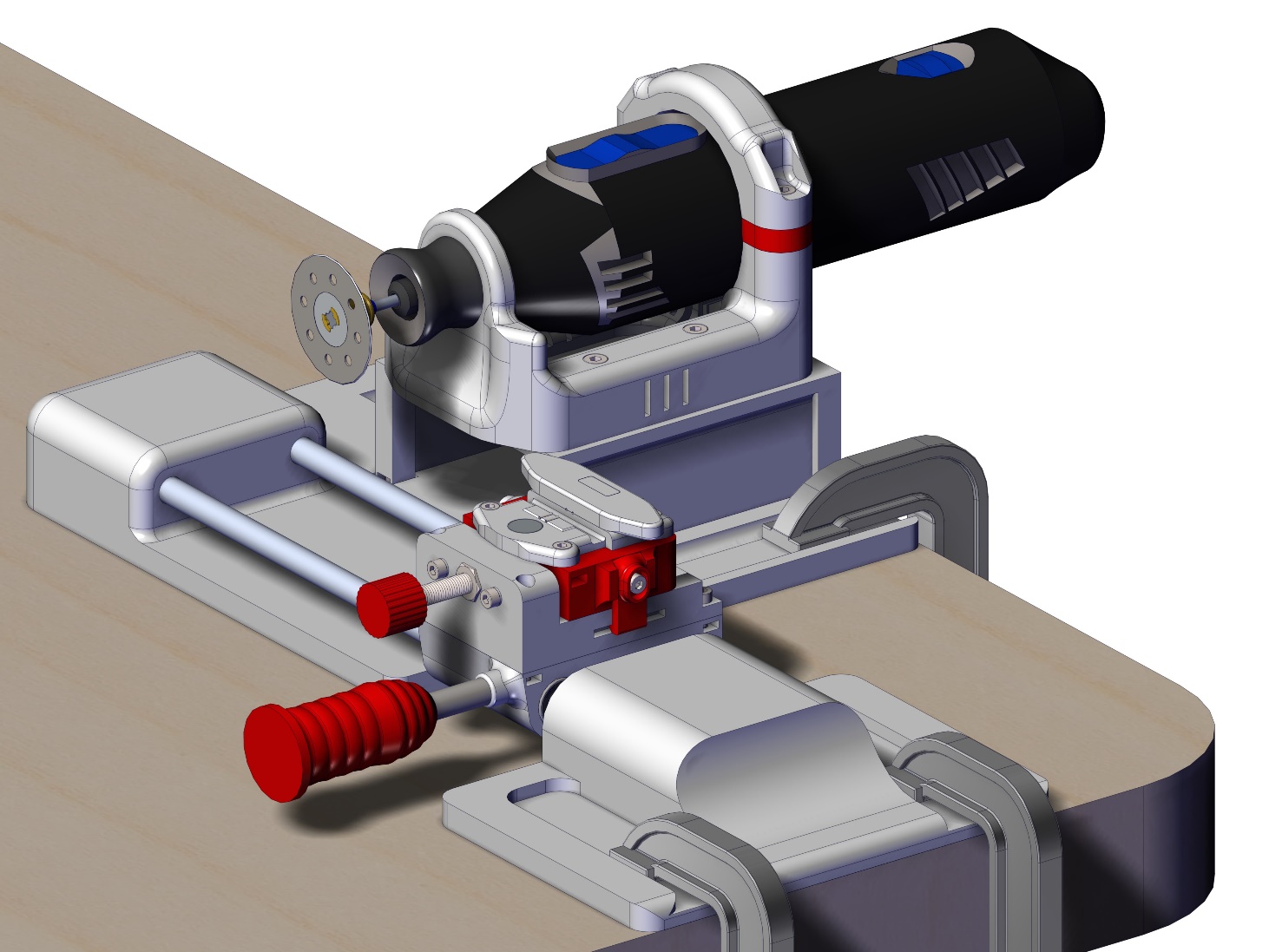


## CAUTION!

## Before considering the realization of this apparatus please contemplate that improper handling of mechanical parts or erroneous electric connections can cause serious injury and damage to you and your property. Please read and understand the instructions below before beginning your project. Follow the instructions, build carefully, and use the appropriate tools. Build at your own risk. We do not take any responsibility for any damage or injury resulting from the assembly or use of this apparatus. You are the manufacturer of your apparatus, and you are responsible for a safe and functioning assembly, usage and maintenance of your setup.

## 1. Bill of Materials

### 1.1 3D printed parts

The complete list of the 3D printed parts is described below. Most of the parts can be printed by an FDM 3D printer with the following settings:

- Nozzle: 0.4 mm
- Material: PLA (ABS or PETG can also be used, even if ABS print of larger models might present plate adhesion difficulties)
- Layer height: 200 µm
- Temperatures: depends on the used material, check your supplier recommendations
- Speed: depends on your 3D printer. We used a customized Prusa i3 variation with direct extrusion at 60 mm/sec.
- Supports: No
- Placement in slicer: when preparing your gcode (or equivalent) it is suggested to place the part in a way that allows the maximum surface area of contact with the printing plate.

Note: some of the parts might require different print settings. Please refer to the description in the Table below.

| **Preview** | **Part Name** | **Print notes:** | **Qty.** |
| --- | --- | --- | --- |
| 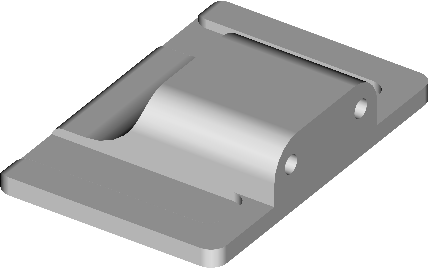 | Bottom rail support | - | 1 |
| 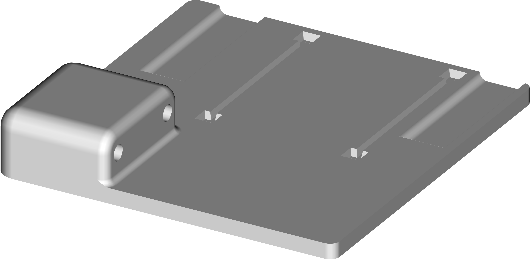 | Top rail support | - | 1 |
| 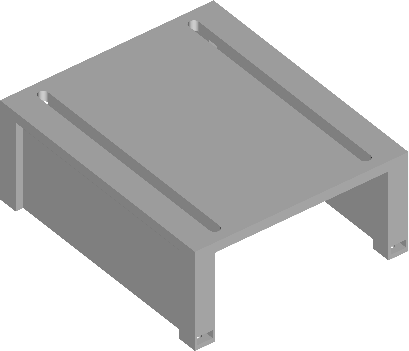 | Multitool positioner | Supports (in grey). Example:  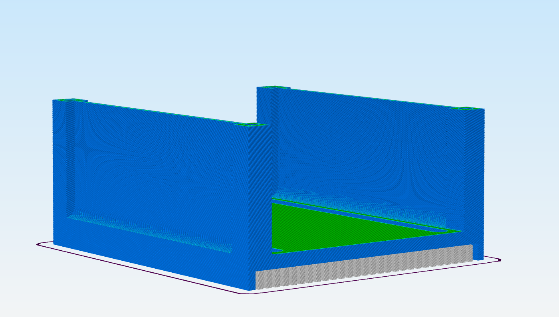 | 1 |
| 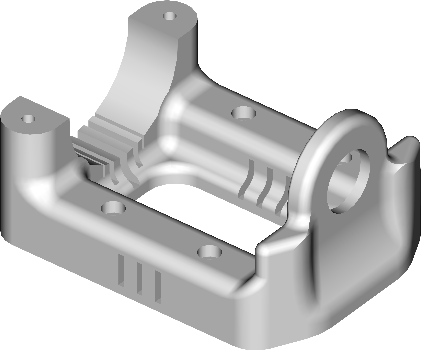 | Multitool support | This support was designed for Dremel 4000. Consider a different design for different multitools. | 1 |
| 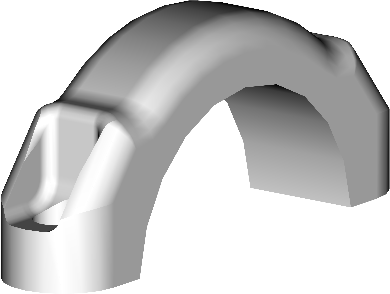 | Multitool tightener | Supports (in grey). Example: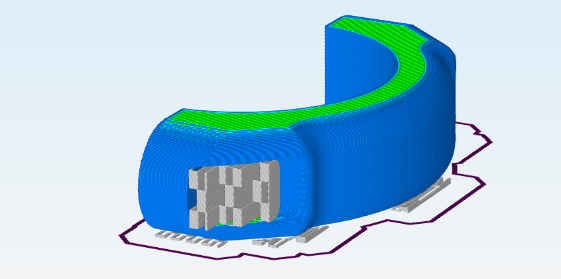 | 1 |
| 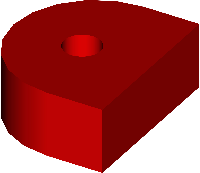 | Multitool spacer | - | 1 |
| 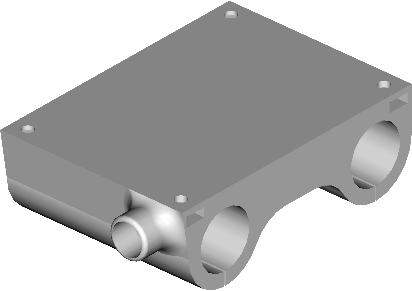 | Base Carriage | Plating suggestion:  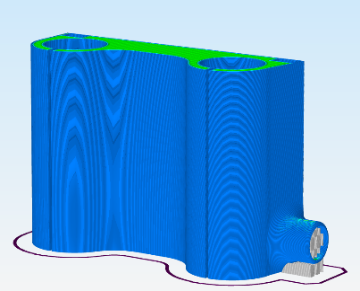 | 1 |

| 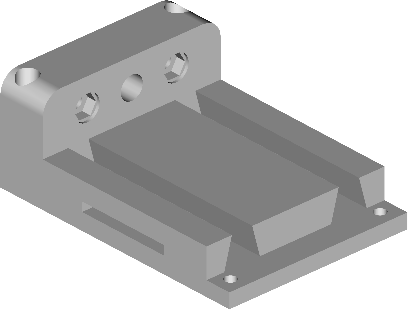 | Length regulator base | - | 1 |
| --- | --- | --- | --- |
| 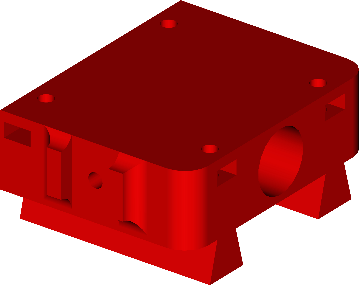 | Length regulator v.2 | - | 1 |
| 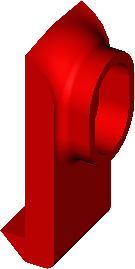 | Centering support | - | 2 |
| 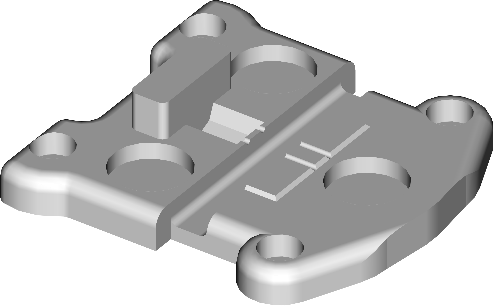 | Cannula support - hippocampus | - | 1 |
| 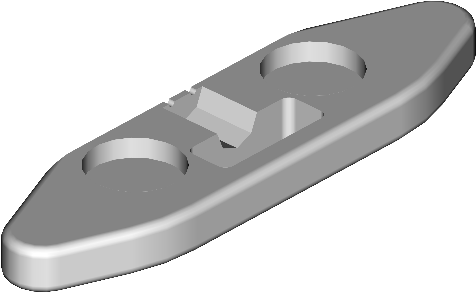 | Cannula support  cover | - | 1 |
| 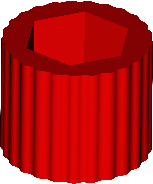 | Cutting length  regulator handle | - | 1 |
| 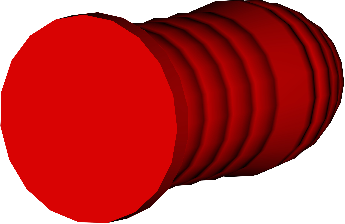 | Carriage handle | - | 1 |

### 1.2 Hardware

| **Preview** | **Part Name** | **Notes** | **Qty.** |
| --- | --- | --- | --- |
| 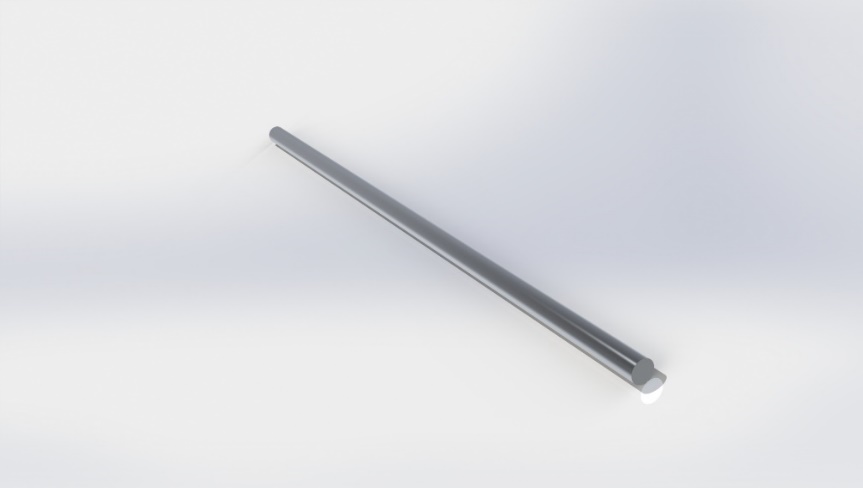 | Stainless steel bar | 270x8Ø mm | 2 |
| 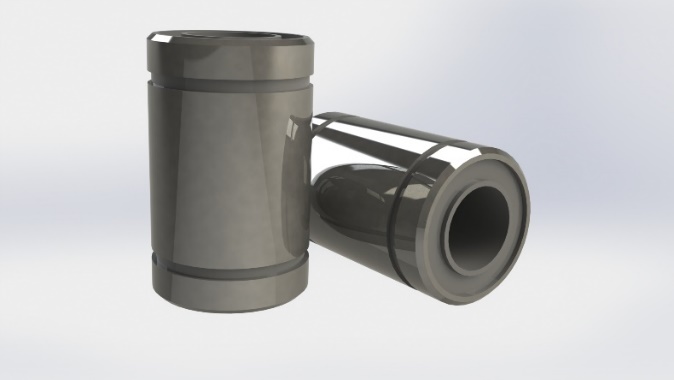 | Linear ball bearing | LM8UU  Ø 8 mm | 3 |
| 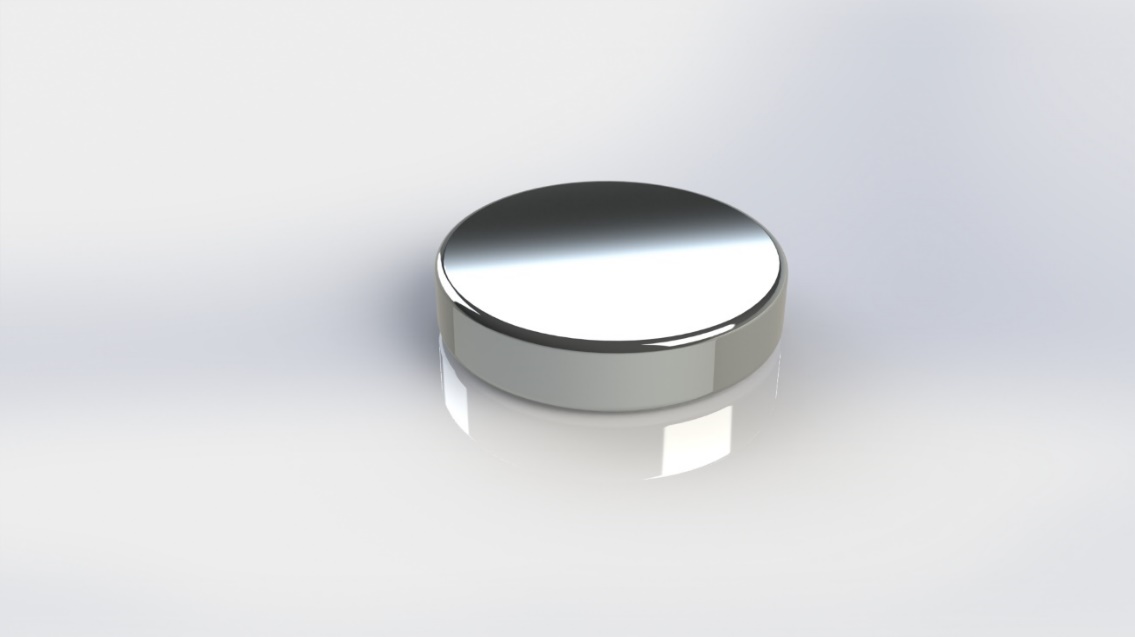 | Neodymium magnet | 10x2 mm | 5 |

| 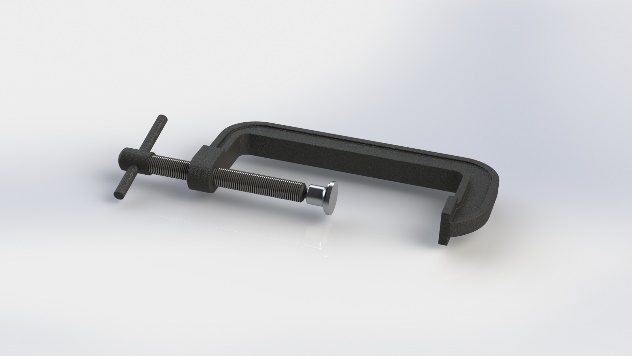 | C-Clamp | Size depending upon the table thickness where you plan to fix the apparatus | 4 | |  |
| --- | --- | --- | --- | --- | --- |
| 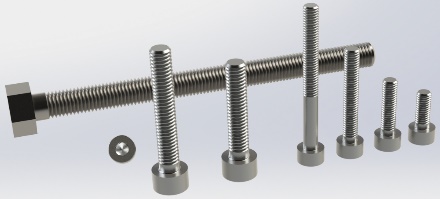 | Screws Hex | M6x80 mm Bolt  M4x25 mm  M4x20 mm  M3x30 mm  M3x20 mm  M3x12 mm  M3x8 mm | 1  4  2  2  2  4  8 | |  |
| 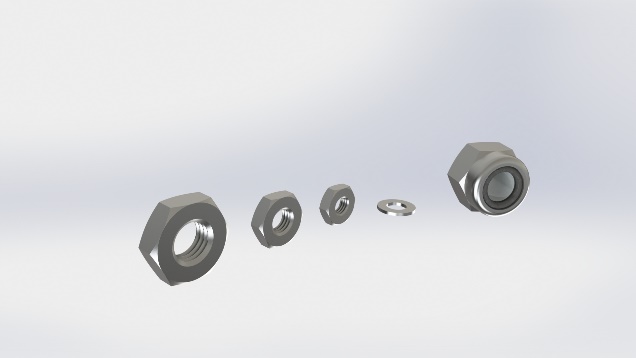 | Nuts and Washers | M3 Nut  M4 Nut  M6 Nut  M6 Locknut  M3 Washer | | 14  4  1  1  2 | |
| 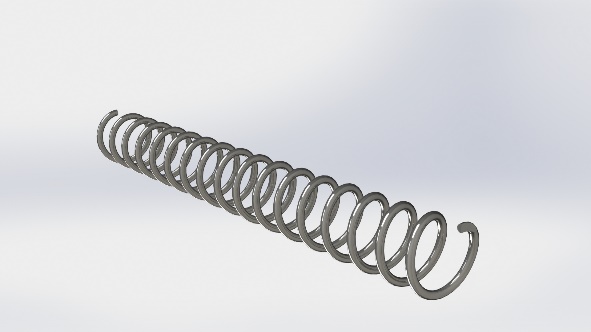 | Spring | - | | 2 | |
| 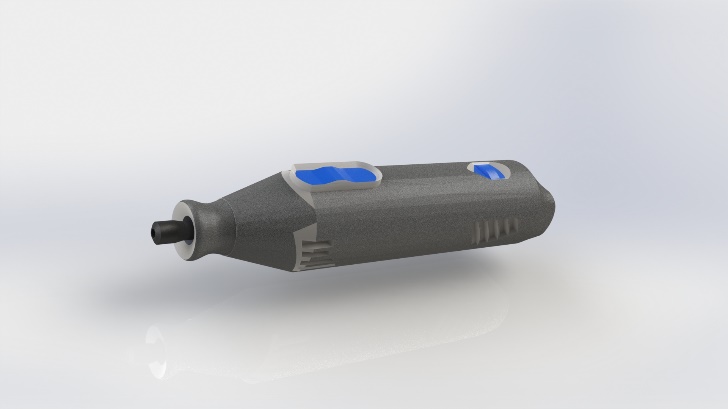 | Multitool | Dremel 4000 | | 1 | |
| 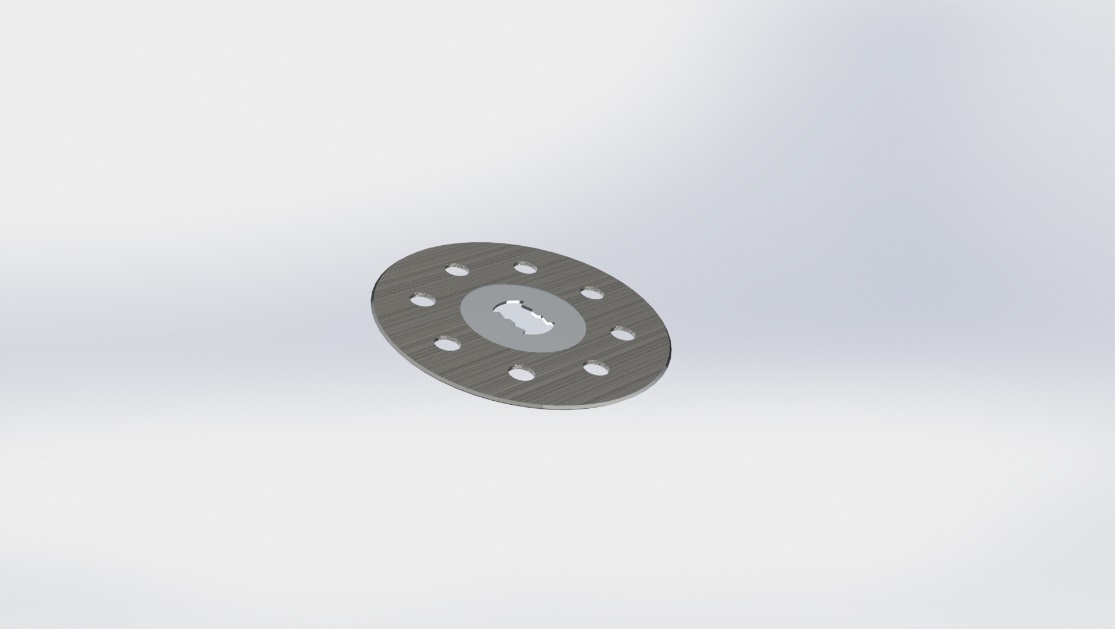 | Blade | - | | 1 | |

| 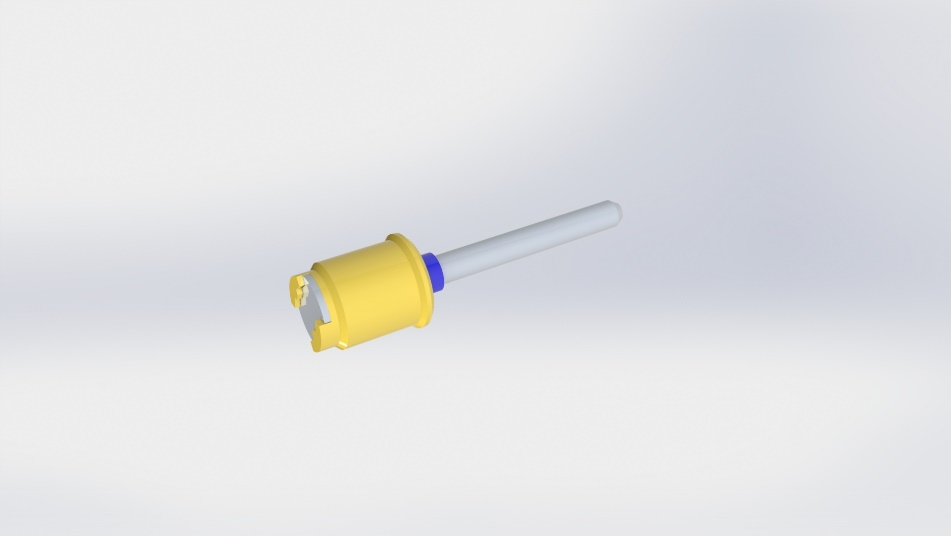 | Blade mount | - | 1 |
| --- | --- | --- | --- |
| 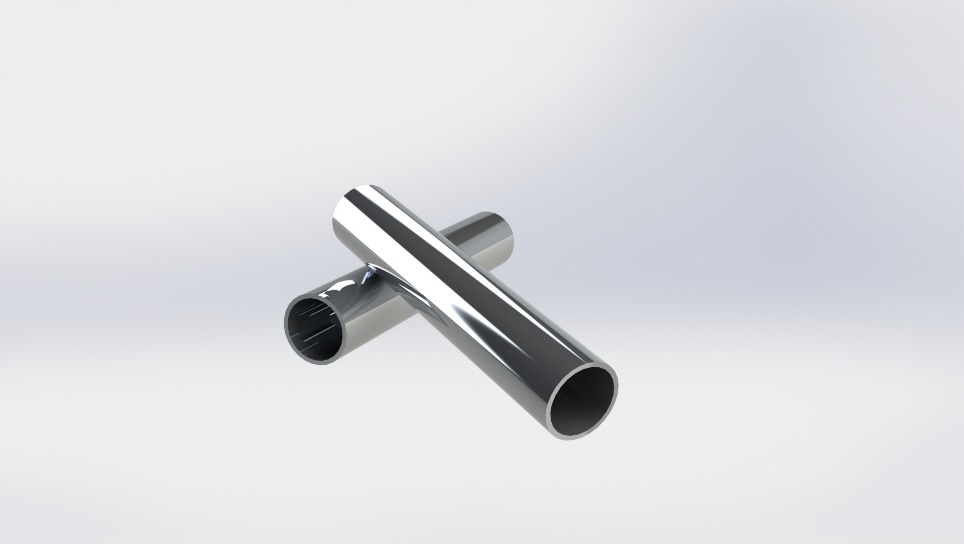 | Guides | - | 2 |
| - | Required  tools | M3 Allen key  M4 Allen key  Flathead screwdriver  Appliance oil | - |

## 2. Step-by-step assembly Instructions

## 2.1 Carriage Assembly


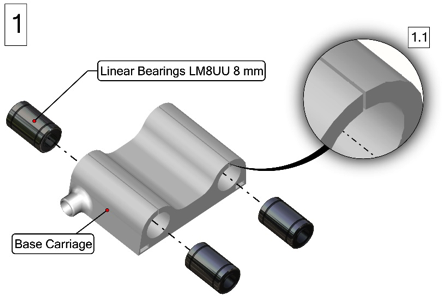


1

**1.** Insert the ball bearings in the base carriage as shown in Figure 1. The space shown in the inset 1.1 can be gently widened with a flathead screwdriver to make a smoother insertion of the Bearings.

**2.** Once inserted, bearings should be positioned as in the cutout view shown in Figure 2.


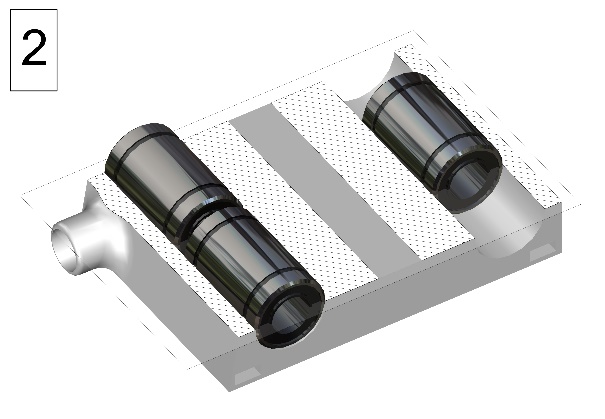


2


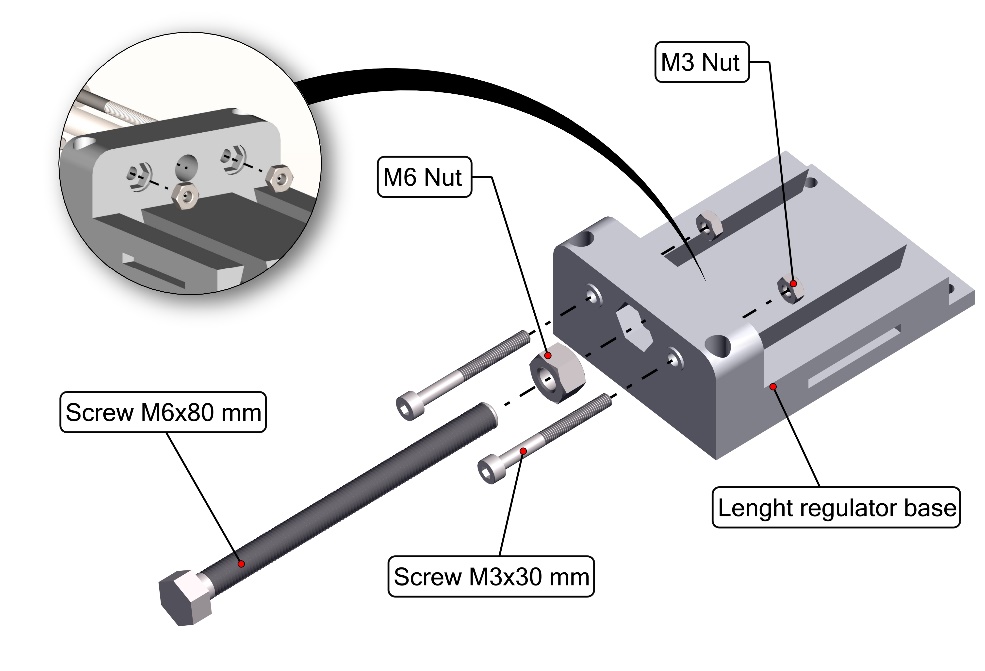


3

**3.** Insert the (2) M3 nuts in their position as shown in Figure 3 inset.

Tighten the (2) M3x30 mm screws all the way in.

Insert the M6 nut into its socket and fix it with some superglue (do not put the glue in the threated part).

**4.** Insert the M6x80 mm screw in the corresponding nut (do not tighten too much, use Figure 4 as a reference).


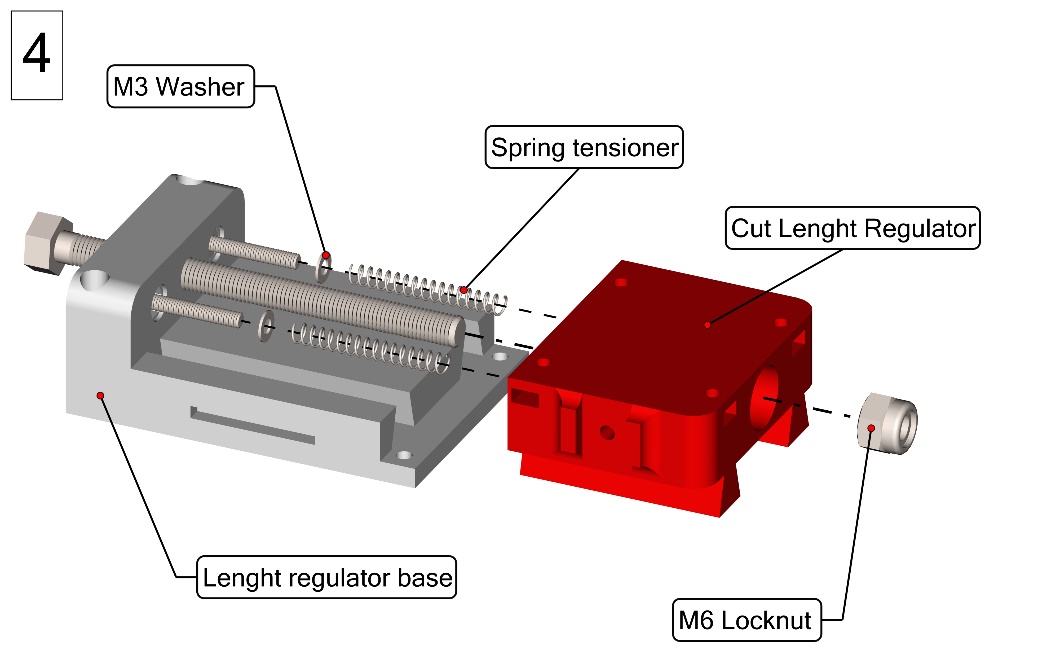


4

Insert the M3 washers in the M3 screw tips.

Insert the spring tensioners.


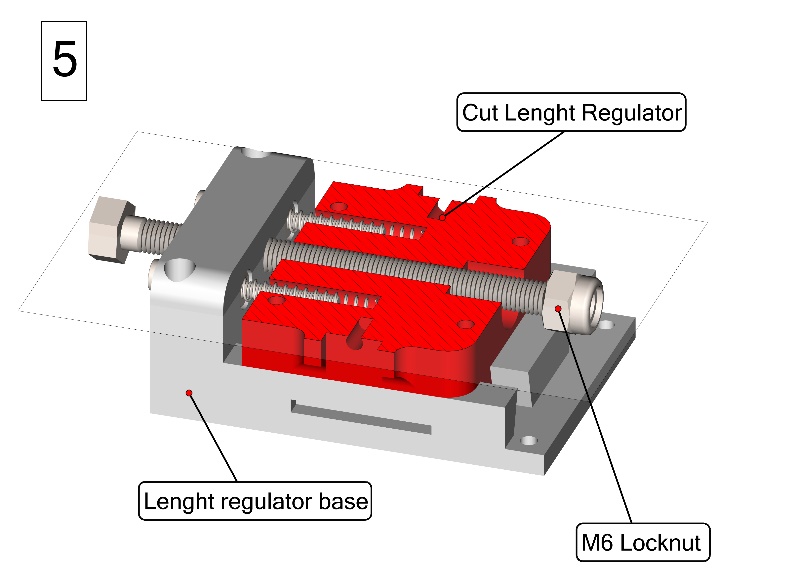


5

**5.** Gently insert the positioner making sure that the spring tensioners and the M6 screw are hosted as shown in Figure 5.

Tighten the self-locking nut to the end of the M6 screw to block the movement of the part as shown in Figure 5.


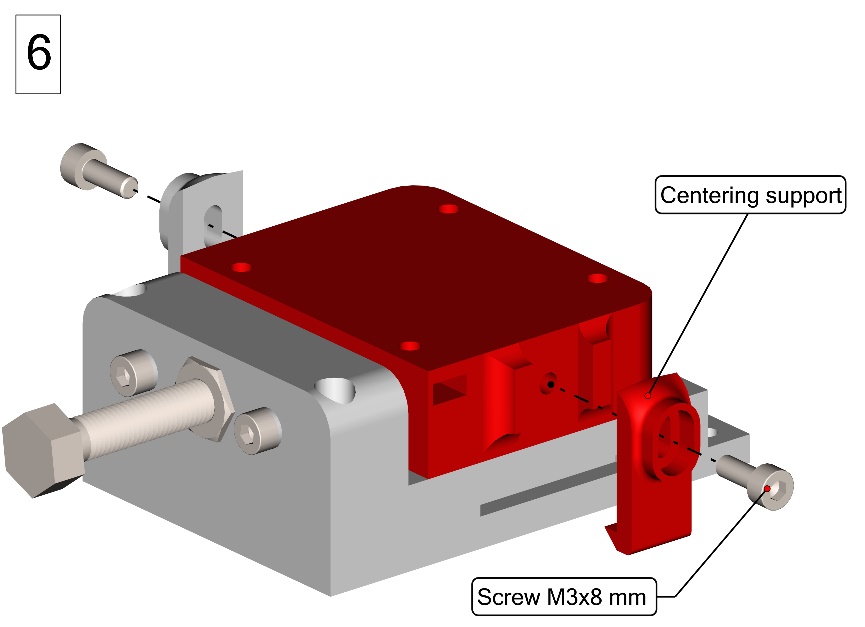


6

**6.** Place the centering supports on both sides of the cut length regulator and tighten (2) M3x8 mm screws into the plastic as shown in Figure 6.

Try to rotate the M6 screw to make sure that its rotation causes the carriage to move back and forth.


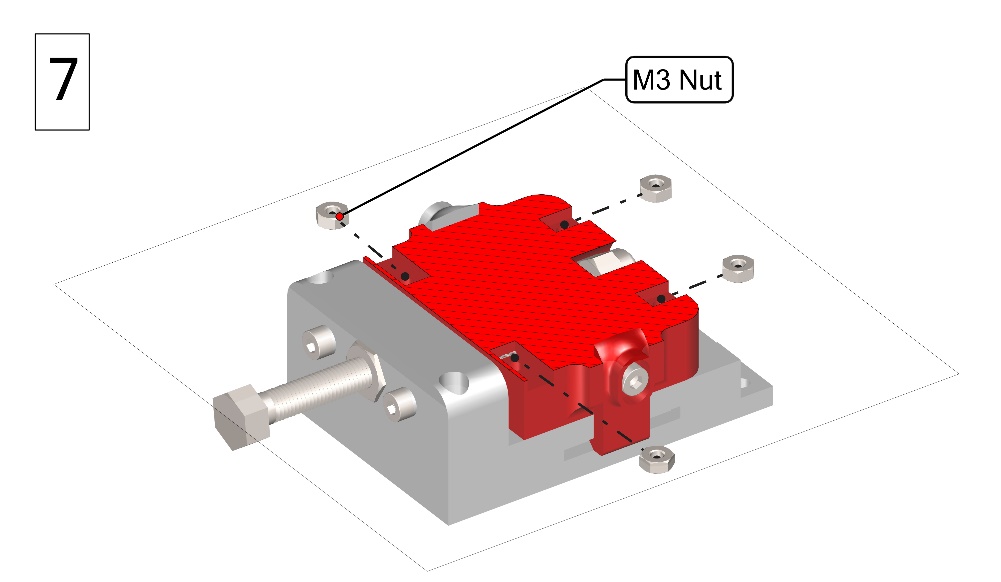


7

**7.** Insert (4) M3 hex nuts in their place as shown in the cutout in Figure 7.

**8.** Insert (4) M3 Nuts in the Base carriage slots as shown in Figure 8.


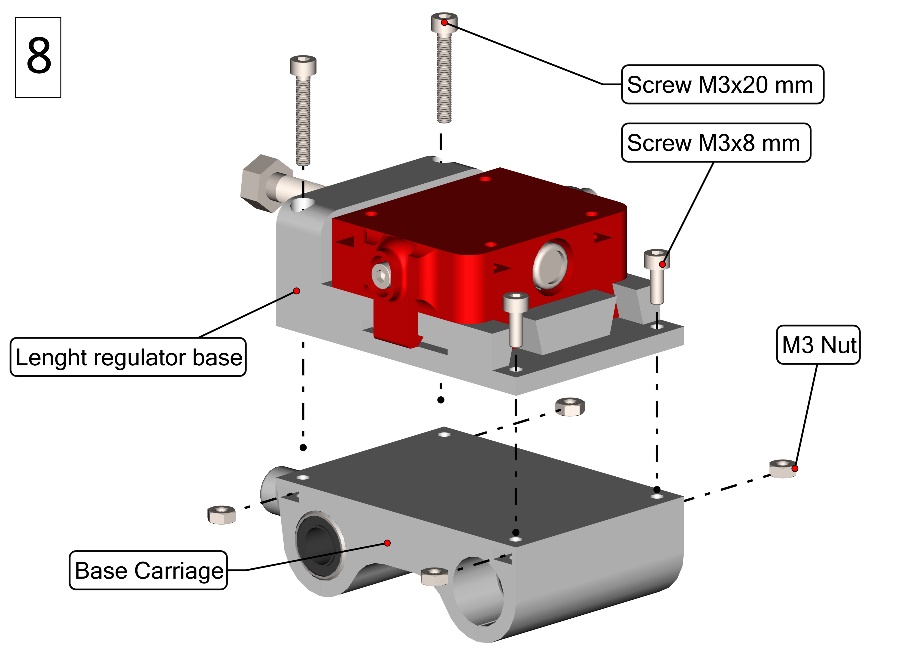


8

Put the Length regulator base on top of the base carriage and insert the (2) M3x20 mm screws and the (2) M3x8 mm screws as shown in Figure 8. Thus, tighten them making sure that the screws enter in the nuts and stabilize the assembly.


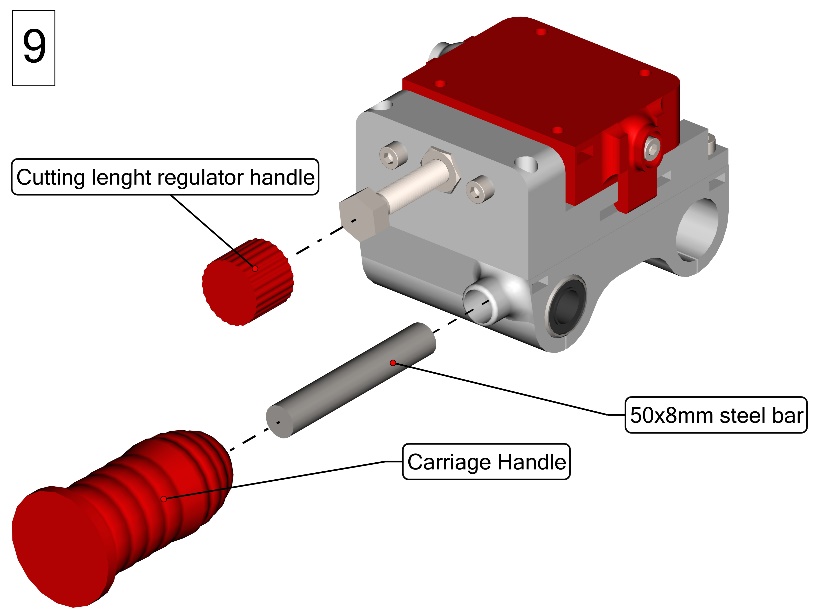


9

**9.** Use superglue to fix the handles and the steel bar as shown in Figure 9.

10.1


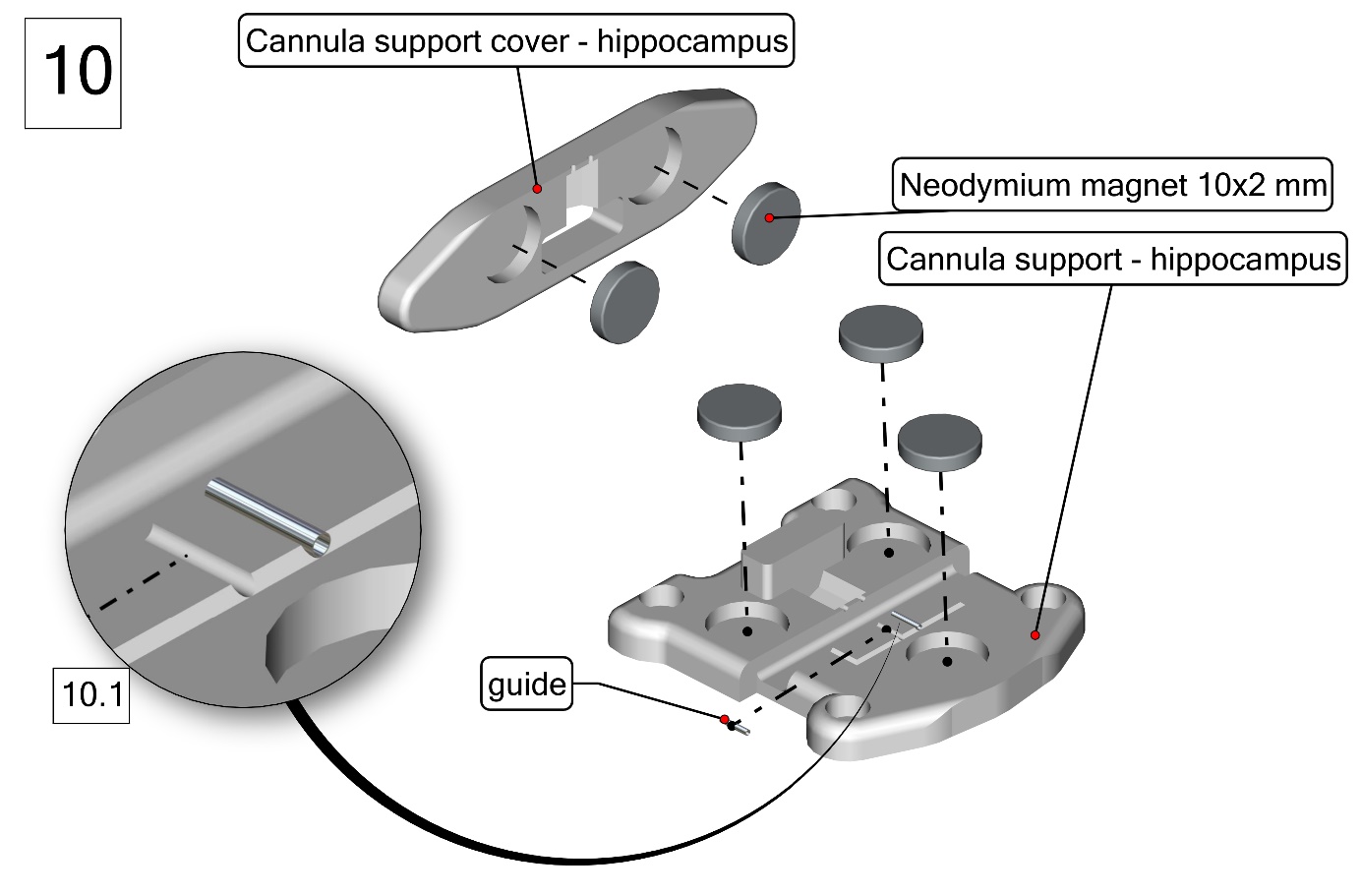


10

**10.** Use a drop of superglue to fix the (5) magnets in position as shown in Figure 10.

**ATTENTION**: make sure that the magnets located in the cannula support and its cover are placed in a way they are attracting themselves.

Use superglue to fix the guides in position as shown in the inset 10.1 (it is advised to use tweezers to correctly locate this part).


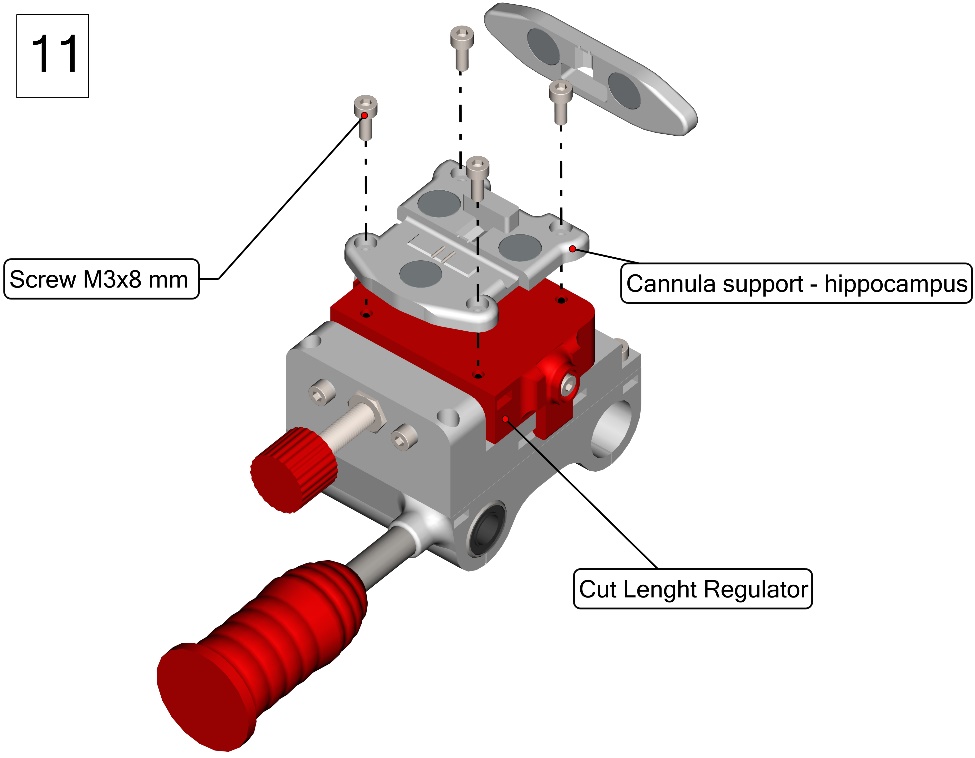


11

**11.** Use (4) M3x8 mm screws to fix the cannula support to the carriage. Make sure that the screws make contact and tighten with the M3 nuts that are present in the carriage (Figure 11).

The carriage is now complete.

## Frame Assembly


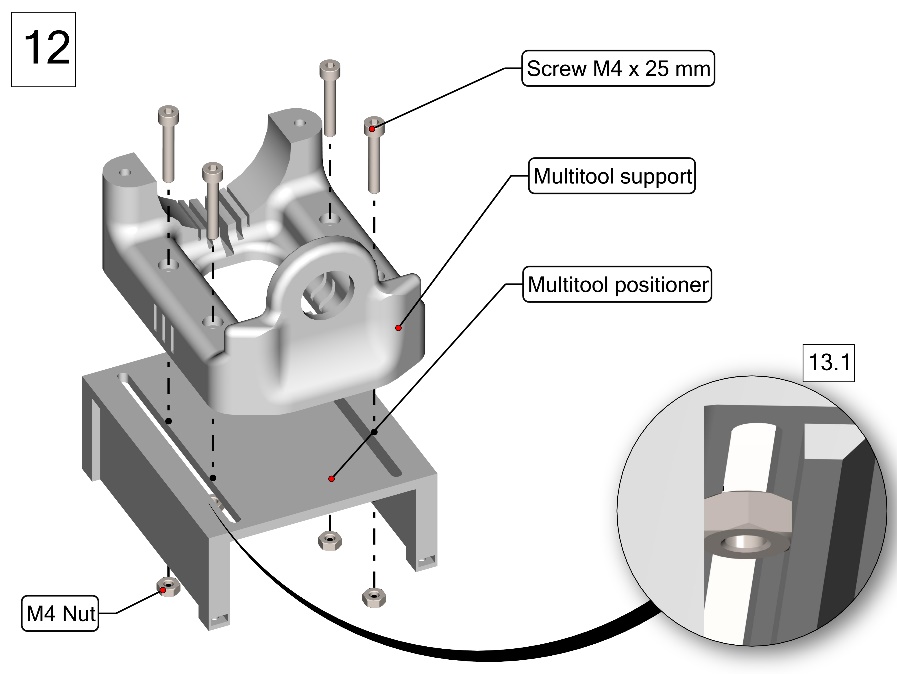


12.1

12

**12.** Fix the Multitool support to the multitool positioner by using 4 M4x25 mm screws.

Do not overtighten the screws, their tension can be regulated later (Figure 12).

Make sure that the nuts are positioned as shown in the inset 12.1.

**ATTENTION**: Remember to check that your multitool is compatible with the mount.


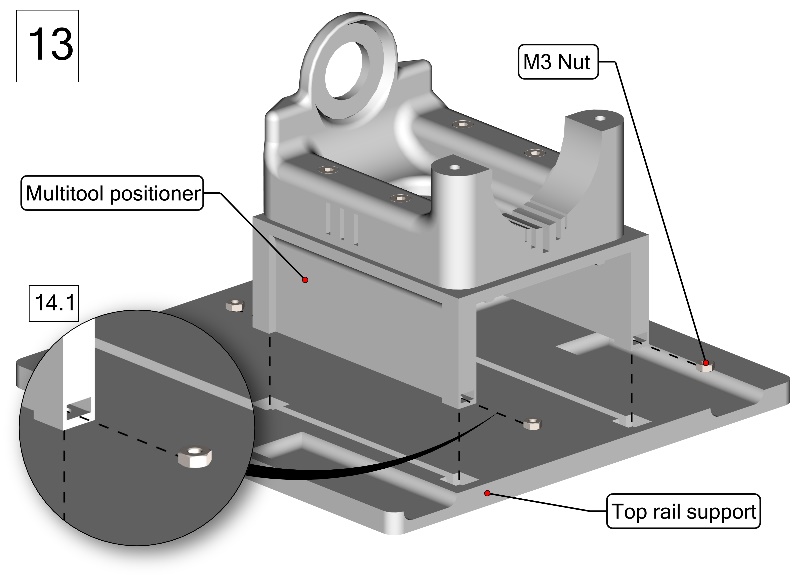


13

13.1

**13.** Insert (4) M3 Nuts in the dedicated slots of the Multitool positioner as shown in Figure 13. A better view of the procedure can be observed in the inset 13.1.

Gently place the positioner in the dedicated slot of the Top rail support.


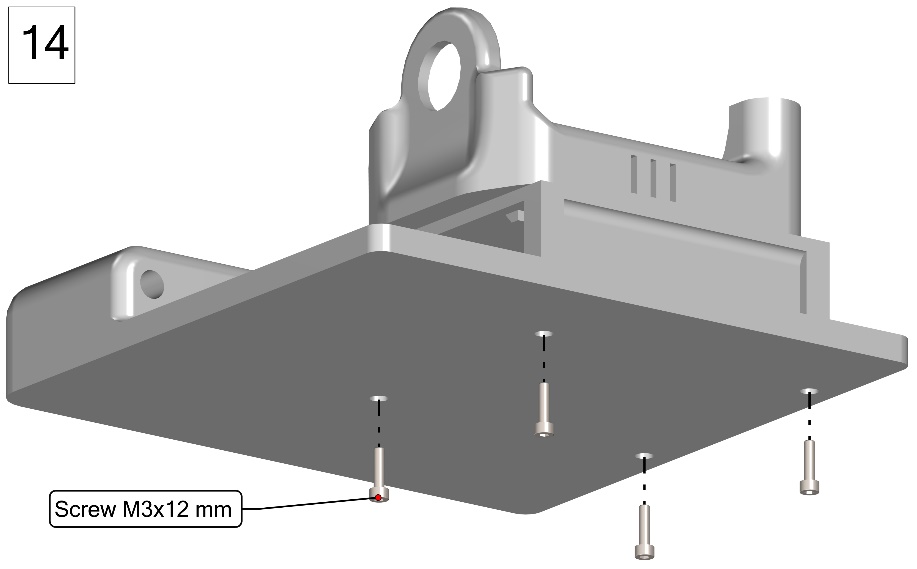


14

**14.** Use (4) M3x12 mm screws to tighten the Top rail support to the multitool positioner (Figure 14).

Fix them firmly, making sure that the screws reach the nuts correctly, and that once tightened the screw is not coming out from the piece.


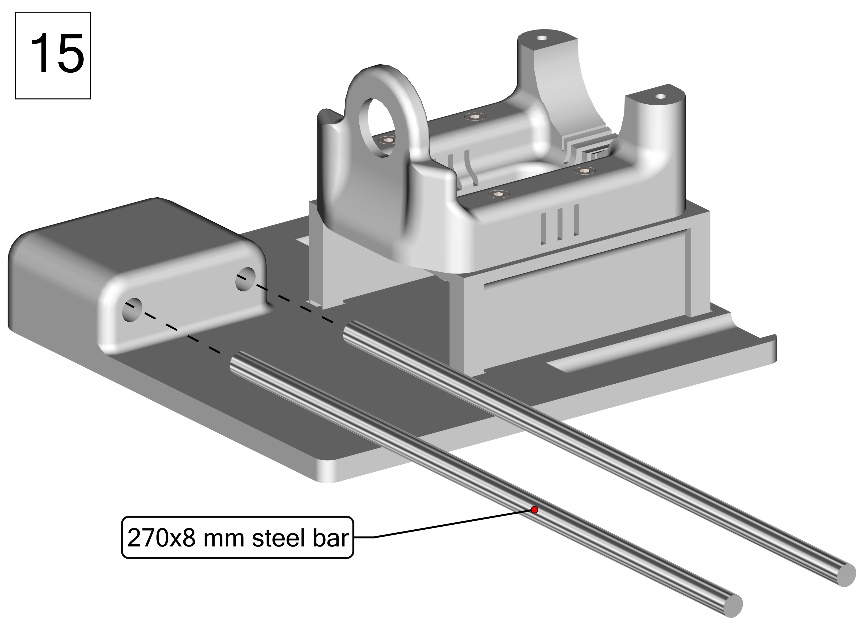


15

**15.** Insert the steel bars in their slots in the top rail support as shown in Figure 15.

*Note that the steel bars might be difficult to insert. In this case some appliance oil can be used to the insertion process.*


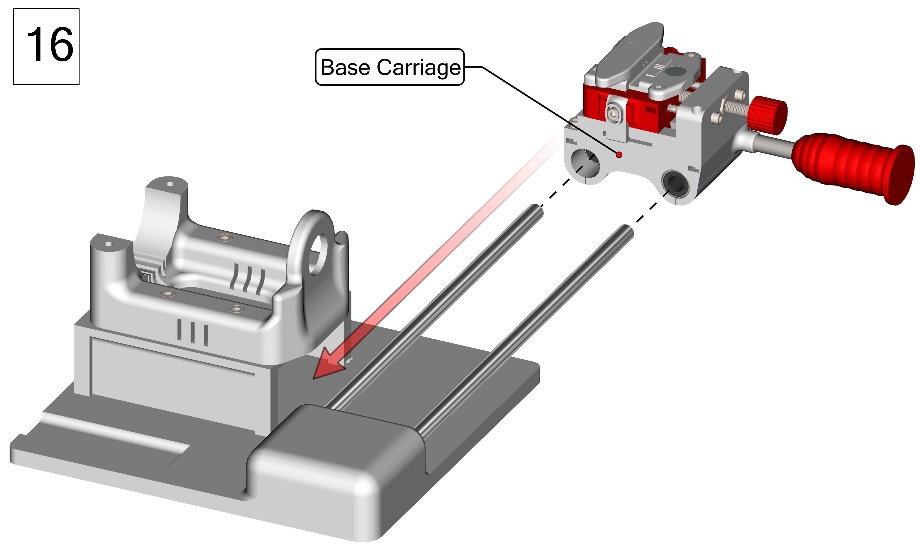


16

**16.** Fix the previously assembled carriage to the steel bars by using the linear bearrings (Figure 16).

**ATTENTION**: Take your time and do this operation as gently as possible to avoid damaging the bearrings.

After the carriage is inserted, push it towards the other end of the rail.


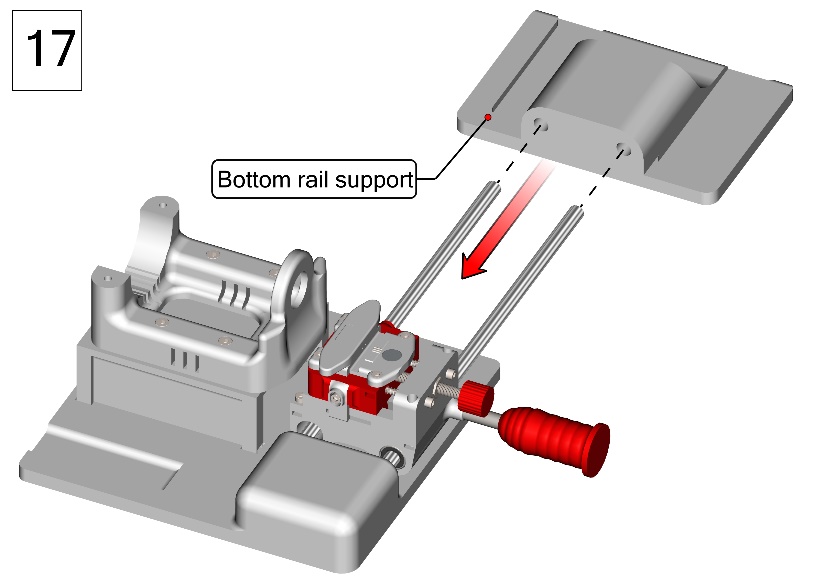


17

**17.** Push the bottom rail support towards the steel bars as indicated in Figure 17.

*The positioning might be difficult, but some appliance oil can be used to smoothen the process.*

**18.** Fix the cutting apparatus to a table corner by using (4) C-Clamps as shown in Figure 18.


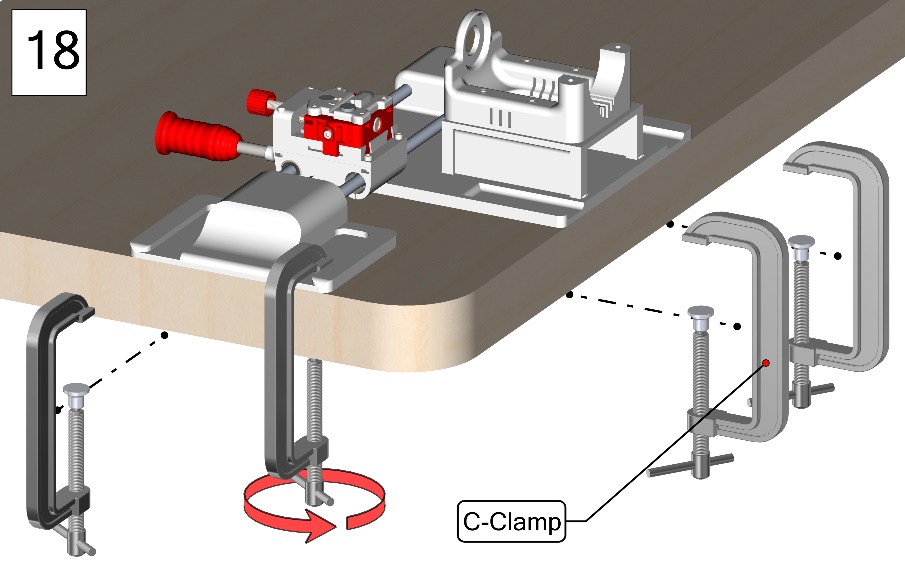


18


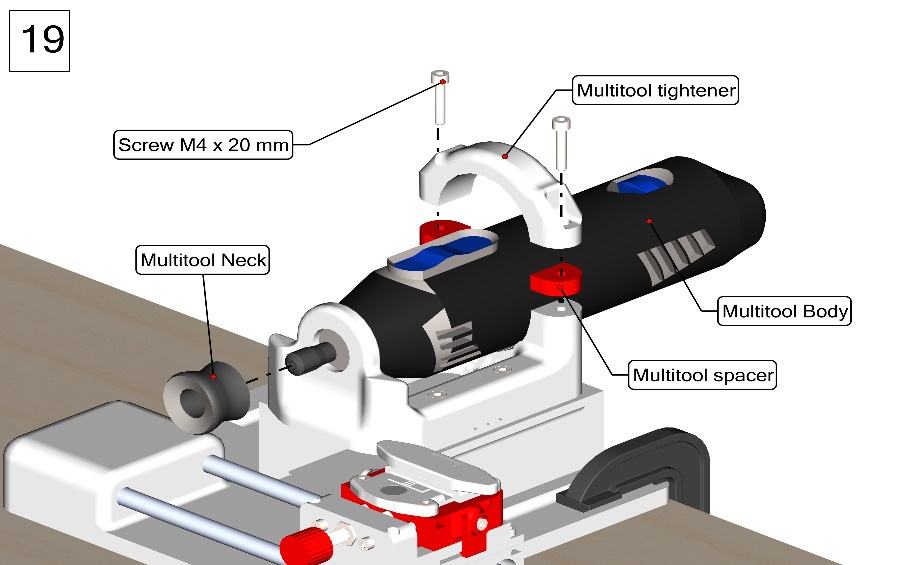


19

**19.** Position the Multitool in its slot on the multitool positioner.

Screw the multitool neck back in place.

Finally, insert and tighten (2) M4x20 mm screws through the multitool tightener and the spacer into the multitool positioner.
