# Supplementary material for: Application of 3D Printing Technology to Produce Hippocampal Customized Guide Cannulas
Source: eNeuro. 2022 Sep 27;9(5):ENEURO.0099-22.2022. doi: 10.1523/ENEURO.0099-22.2022 (PMC9522464; doi:10.1523/ENEURO.0099-22.2022)
Supplement: Figure 2-1 — *.Stl files, *.STEP files, and technical drawings. Download Figure 2-1, ZIP file. [file enu-eN-MNT-0099-22-s02.zip › Technical drawings/7_Multitool support rev2.PDF]

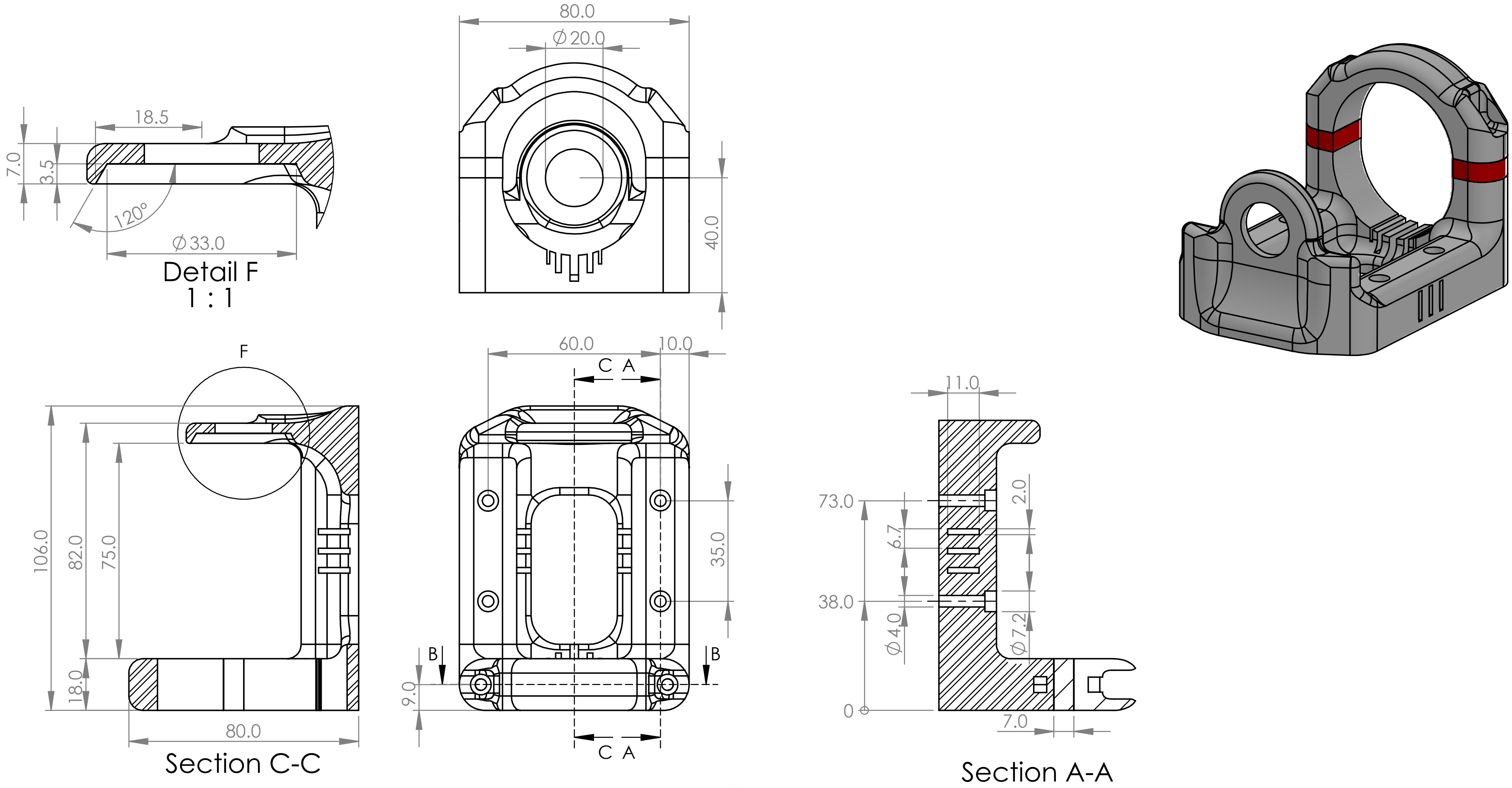

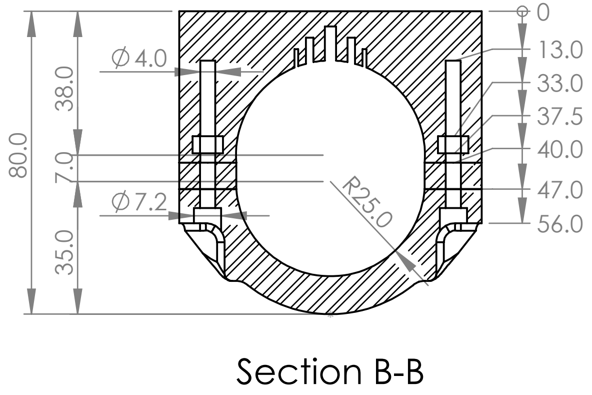

MODEL FILE: MULTITOOL SUPPORT	
DIMENSIONS: mm	SCALE: 1:2
MATERIAL: PLA	DRAWING Nº: 7
AUTHOR: D.Pi/W.G.	NOTES: Only relevant quotes are indicated
