# Supplementary figures and images for: Application of 3D Printing Technology to Produce Hippocampal Customized Guide Cannulas
Source: eNeuro. 2022 Sep 27;9(5):ENEURO.0099-22.2022. doi: 10.1523/ENEURO.0099-22.2022 (PMC9522464; doi:10.1523/ENEURO.0099-22.2022)

3

2

1

C

C

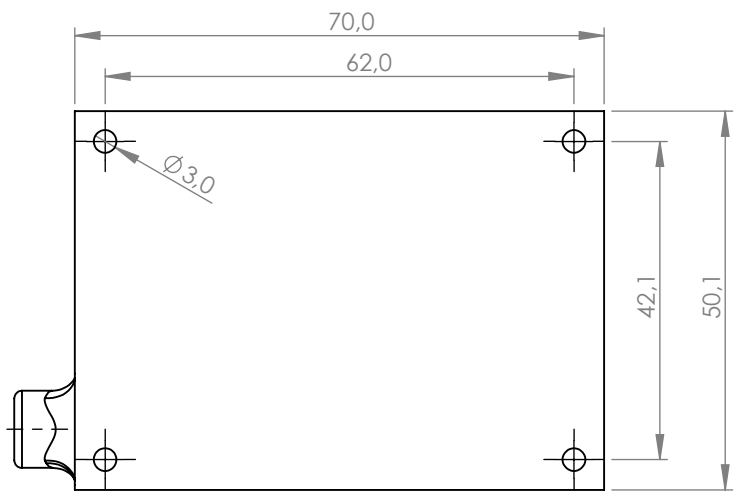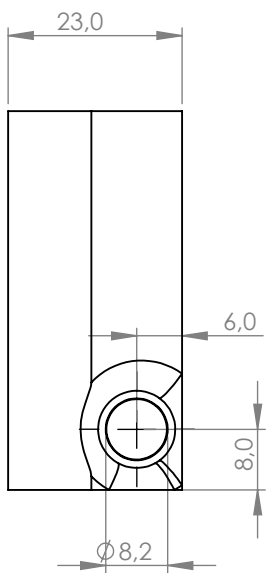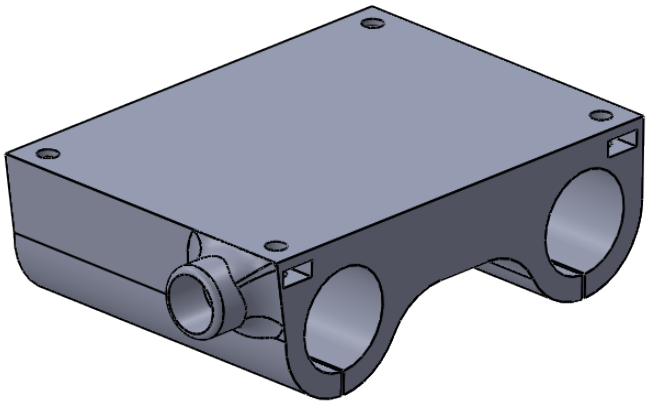

B

B

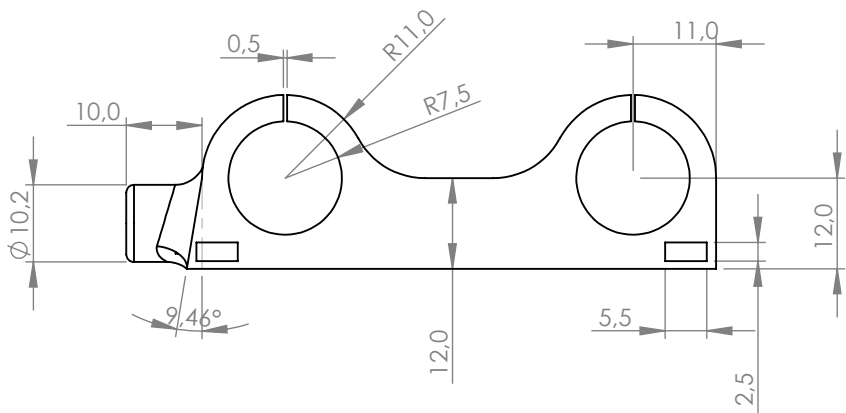

MODEL FILE:

BASE CARRIAGE

DIMENSIONS:

mm

SCALE:

1:2

MATERIAL:

PLA

DRAWING N°:

4

AUTHOR:

D.Pi/W.G.

NOTES:

3

2

1

A

A

Supplement: Figure 2-1 — *.Stl files, *.STEP files, and technical drawings. Download Figure 2-1, ZIP file. [file enu-eN-MNT-0099-22-s02.zip › Technical drawings/4_Base Carriage.PDF]

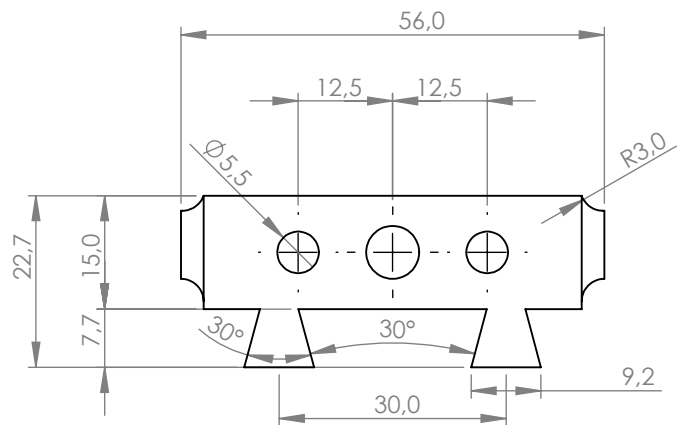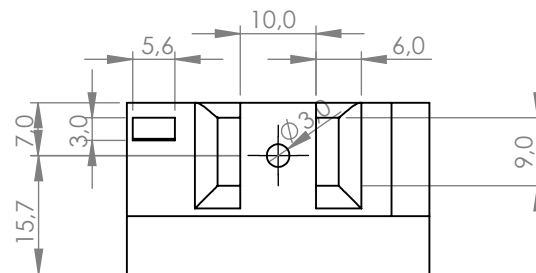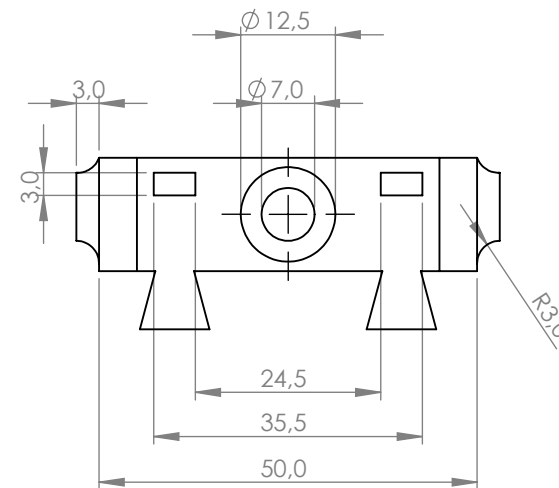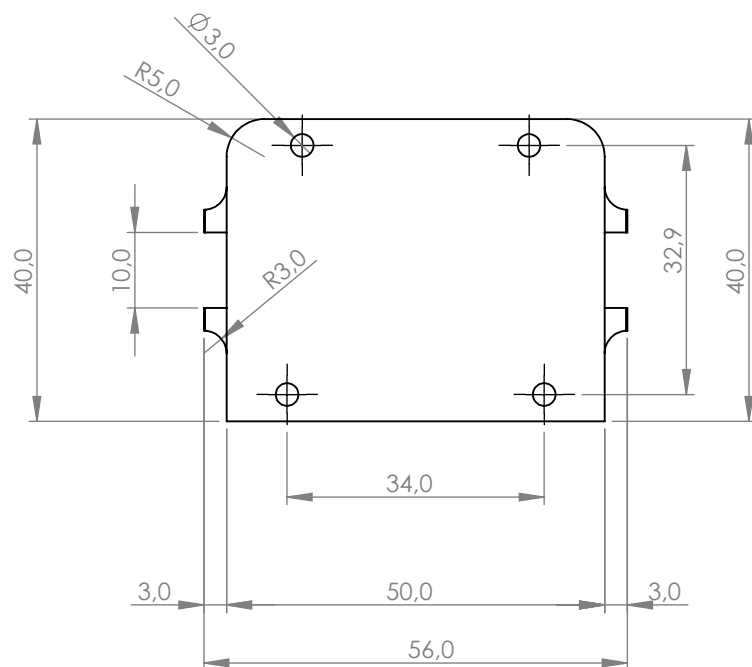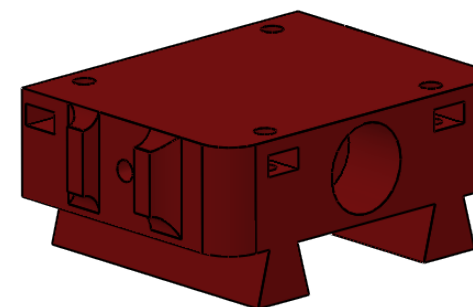

MODEL FILE:  
CUT LENGHT REGULATOR

DIMENSIONS: mm SCALE: 1:2

MATERIAL: PLA DRAWING N°: 6

AUTHOR: D.Pi/W.G.

NOTES:

Supplement: Figure 2-1 — *.Stl files, *.STEP files, and technical drawings. Download Figure 2-1, ZIP file. [file enu-eN-MNT-0099-22-s02.zip › Technical drawings/6_Cut lenght regulator.PDF]
